# A Low‐Temperature Solar Salt Approach to Fabricate Crystalline Polymeric Carbon Nitride for H_2_O_2_ Efficient Photosynthesis

**DOI:** 10.1002/advs.202512549

**Published:** 2025-10-13

**Authors:** Jie Tang, Junqing Li, Lu Li, Chengying Xu, Hui Yang, Chao Chen, Weiqiang Hao, Yi Yang, Kelin He, Linfu Xie, Feng Tang, Zimo Huang, Qitao Zhang

**Affiliations:** ^1^ Changzhou Vocational Institute of Engineering Changzhou 213000 China; ^2^ International Collaborative Laboratory of 2D Materials for Optoelectronics Science and Technology of Ministry of Education Institute of Microscale Optoelectronics Shenzhen University Shenzhen 518060 China; ^3^ College of Environmental Science and Engineering Yangzhou University Yangzhou 225127 China; ^4^ School of Metallurgy and Environment Central South University Changsha 410083 China; ^5^ School of Chemistry and Chemical Engineering Yangzhou University Yangzhou 225002 China

**Keywords:** exciton dissociation, H_2_O_2_ photosynthesis, photocatalyst, polymeric carbon nitride, solar salt

## Abstract

Photocatalytic technology based on polymeric carbon nitride (PCN) offers a sustainable and ecofriendly approach to hydrogen peroxide (H_2_O_2_) production field. Nonetheless, the effectiveness of PCN is significantly hindered by the strong binding energy of excitons and slow transfer ability of carriers. Herein, SS‐UPCN‐375 photocatalyst is prepared by one‐pot solar salt (60% NaNO_3_–40% KNO_3_) thermal polymerization at 375 °C for the first time using nitrogen‐rich precursors. The use of solar salt as a thermal reaction medium facilitates rapid control of the crystallization process and the electronic structure of photocatalysts, and yielding SS‐UPCN‐375 characterized by high crystallinity, augmented visible light utilization, and efficient exciton dissociation capability. Most importantly, SS‐UPCN‐375 demonstrates outstanding H_2_O_2_ artificial photosynthesis through two‐step single‐electron oxygen reduction reaction pathways, and achieves an impressive H_2_O_2_ production rate of 1.80 mmol L^−1^ h^−1^, which is almost 6.7 times superior to that of pristine UPCN. In short, a novel approach that employs solar salt as a low‐temperature solvent to specifically tailor the grain boundary structure and chemical composition of PCN is presented, and it further offers essential guidance for designing high‐performance PCN‐based photocatalysts to promote H_2_O_2_ artificial photosynthesis.

## Graphical abstract

This work initiates a novel low‐temperature solar salt approach to fabricate crystalline polymeric carbon nitride (PCN) photocatalyst. The solar salt can facilitate the rapid crystallization processes and precisely regulate the electronic structure of PCN. The yielded PCN photocatalyst is with high crystallinity, augmented optical utilization, fast exciton dissociation and efficient charge separation, achieving excellent performance for H_2_O_2_ artificial photosynthesis.

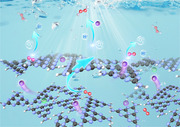

## 1 Introduction

Hydrogen peroxide (H_2_O_2_) is one of the cornerstone compounds used in the contemporary chemical engineering industry because of the pivotal role it plays across a variety of energy and environmental applications.^[^ ^1^, ^2^ ^]^ The industrial production of H_2_O_2_ primarily relies on the traditional anthraquinone process, which is typically regarded as the significant energy consumption and the generation of various chemical wastes, also resulting in considerable environmental pollutions.^[^ ^3^ ^]^ Additionally, the direct synthesis of H_2_O_2_ can be achieved using precious metals, such as Pd, Au, and Pt, as catalysts and H_2_/O_2_ mixtures as reactants.^[^ ^4^, ^5^ ^]^ However, these methods have been impeded by multiple drawbacks, including high costs, low product selectivity, complex reaction processes, and inherent danger, which collectively hinder its widespread applications. Therefore, developing an efficient and sustainable H_2_O_2_ preparation technology is of paramount importance. Drawing inspiration from plant photosynthesis, the ability to employ photocatalysts to harness solar energy and facilitate the reaction between water and oxygen for H_2_O_2_ production represents a novel, sustainable, and ecofriendly approach.^[^ ^6^, ^7^, ^8^, ^9^ ^]^ However, the most pressing issue at present involves the myriad challenges associated with the developed photocatalysts, including the following: high cost of preparation, inefficient exciton dissociation, high carrier recombination rate, low utilization of visible light, and low product selectivity.^[^ ^10^, ^11^, ^12^ ^]^ Consequently, the primary avenue toward achieving sustainable H_2_O_2_ production lies in the development of a cost‐effective photocatalyst.

PCN semiconductor materials, distinguished by their metal‐free composition, adjustable electronic properties, suitable band structures, and simple synthesis, hold significant potential for photocatalytic H_2_O_2_ production. Typically, PCN directly synthesized through thermally induced polymerization under high temperature (≥ 500 °C) often exhibits bulk morphology and significant defects, including poor crystallinity, weak light absorption, and low activity, which primarily are attributed to disordered local structures and high exciton binding energy, leading to pronounced exciton effects among carriers.^[^ ^13^, ^14^ ^]^ Thus, the pivotal scientific challenge lies in efficiently achieving exciton dissociation and enhancing the separation and transportation rates of photoexcited charge carriers. Recently, the molten salt method (MSM)^[^ ^15^, ^16^ ^]^ and salt‐template method (STM)^[^ ^17^, ^18^ ^]^ were employed using monomers (melamine, urea, thiourea, dicyandiamide, etc.) and alkali halide salts (e.g., NaCl/KCl/LiCl, KBr/LiBr, etc.) to modulate the intermediate polymerization processes of PCN, thereby enhancing its polymerization and crystallization through solvation and coordination effects. Nevertheless, the drawbacks of these methods include harsh experimental conditions, high energy consumption and low photocatalytic activity of the resulting products. For instance, the preparation temperatures often exceed 500 °C and commonly utilized lithium salts, such as LiCl and LiBr are incompatible with water and oxygen.^[^ ^19^, ^20^ ^]^

Recognized as a cost‐effective heat absorption and storage medium, binary mixed molten salt (60% NaNO_3_‐40% KNO_3_), commonly referred to as solar salt, has been used extensively in the field of photothermal power generation.^[^ ^21^ ^]^ Among the variety of molten salts, solar salt is distinguished by its superior high‐temperature heat transfer capability, low melting point, low cost, high heat flux density, and straightforward post‐processing.^[^ ^22^ ^]^ The employment of solar salt as a reaction medium for synthesizing PCN photocatalytic materials is rarely reported. In this study, it was discovered that solar salt (60% NaNO_3_‐40% KNO_3_) was an effective substitute for traditional molten salts (NaCl/KCl/LiCl, KBr/LiBr). High‐crystallinity PCN could be produced in bulk at a low temperature range of 325 °C to 400 °C through a solid‐state reaction using solar salt as the reaction medium, denoted as SS‐UPCN‐T (denoted as urea‐derived PCN under solar salt at a specific temperature). This novel low‐temperature molten salt approach not only enhanced the crystallinity of PCN but also facilitated alkali ions doping at interstitial sites within the PCN structure, markedly altering the intrinsic properties of the PCN framework. Compared with the pristine PCN, SS‐UPCN‐T samples exhibited larger dipole moments, which promoted their polarization to produce a higher dielectric constant (*ε*_r_) and thus a lower exciton binding energy.

Herein, SS‐UPCN‐T synthesized through the low‐temperature solar salt method was reported, and it demonstrated substantial photocatalytic activity for H_2_O_2_ artificial photosynthesis. Subsequently, SS‐UPCN‐T was characterized by employing a series of advanced spectroscopic techniques, including Kelvin probe force microscopy (KPFM), temperature‐dependent photoluminescence (TD‐PL), femtosecond ultrafast transient absorption (fs‐TAS) and electron spin resonance (ESR), as well as density‐functional theory (DFT) calculations. The results showed that the solar salt low‐temperature approach effectively regulated PCN crystallinity and dielectric constant, facilitated exciton dissociation and charge separation efficiency, ultimately augmenting O_2_ adsorption and activation for H_2_O_2_ photosynthesis.

## 2 Results and Discussion

### 2.1 Morphology and Microstructure Characterization

**Figure 1:**
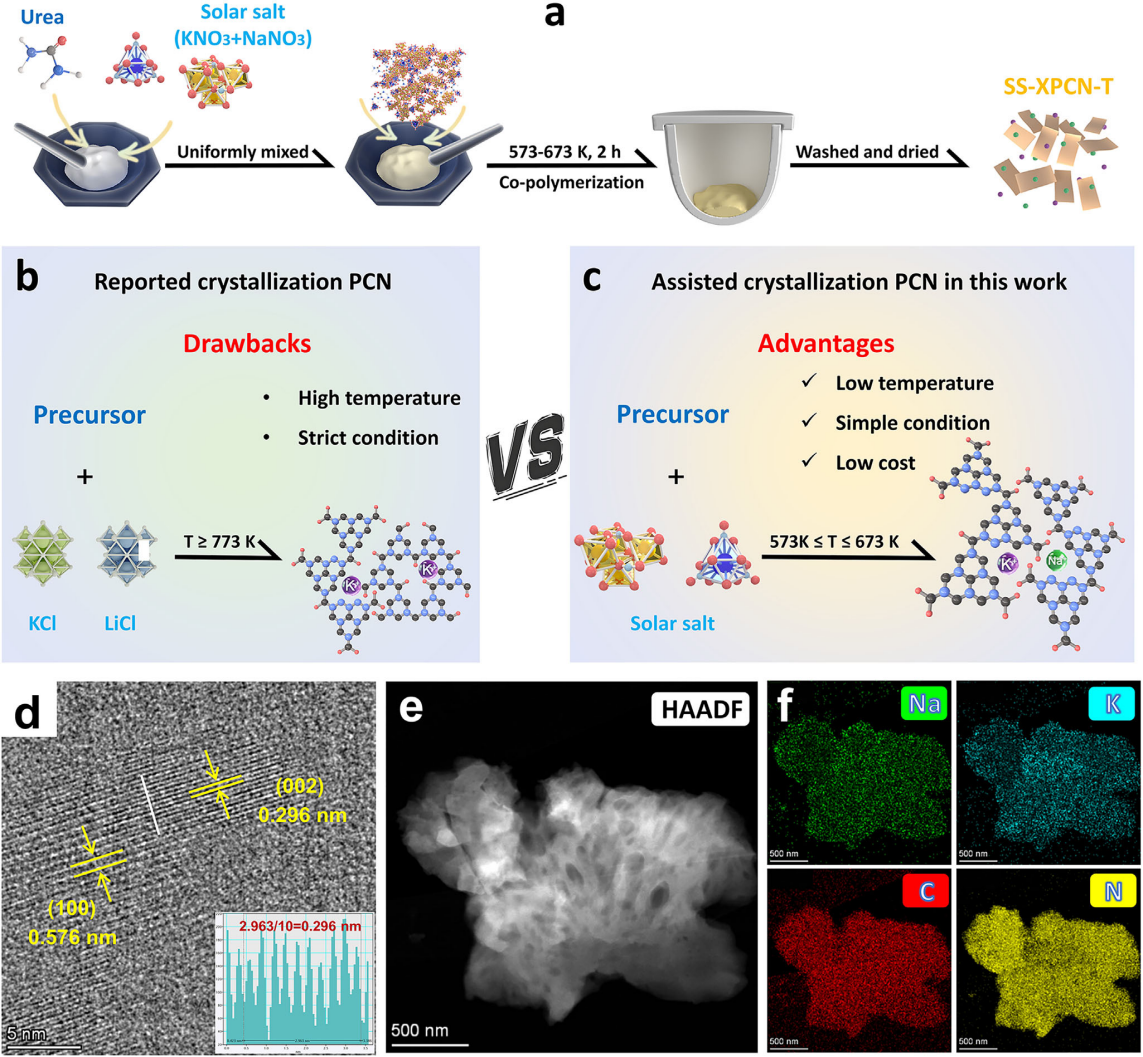
Diagram of a–c) the preparation process for SS‐UPCN‐T, d,e) HR‐TEM images of SS‐UPCN‐375, and f) the HAADF‐STEM and element mapping of SS‐UPCN‐375. The inset in (d) is the lattice fringes of SS‐UPCN‐375 along the white line.

As shown in **Figure** **1**a, SS‐UPCN‐375 photocatalyst with a 2D layered structure was successfully prepared according to a simple low‐temperature solar salt thermal treatment method. Compared with the reported conventional molten salts method (e.g., NaCl/KCl/LiCl/KBr/LiBr; Figure 1b), this method possesses the advantages of low cost, simple operation and low reaction temperature (Figure 1c). Furthermore, the microstructural morphology of all the synthesized materials was investigated using TEM and SEM technologies. As depicted in Figure 1d–f and Figures S1 and S2 (Supporting Information), pristine UPCN displayed a typical amorphous structure, and a distinctly wrinkled and irregularly folded structural morphology of PCN‐based photocatalysts was revealed in Figure S2 (Supporting Information). However, further HR‐TEM analysis of the prepared SS‐UPCN‐375 sample exhibited distinct structural features, enhanced local order and higher crystallinity. The average lattice fringe distances in SS‐UPCN‐375, measured at 0.576 and 0.296 nm, corresponding to the repeating packed distance of the heptazine units (100) and the irregularly ordered graphitic layer stacking distance (002), respectively, which suggested that SS‐UPCN‐375 presented high local order and crystallinity when compared with UPCN (Figure 1d). HAADF‐STEM mapping images of SS‐UPCN‐375 further indicated that C, N, Na, and K elements were uniformly distributed across the entire area of the catalyst's framework (Figure 1e,f).

### 2.2 Phase Composition and Photoelectronic Properties

**Figure 2:**
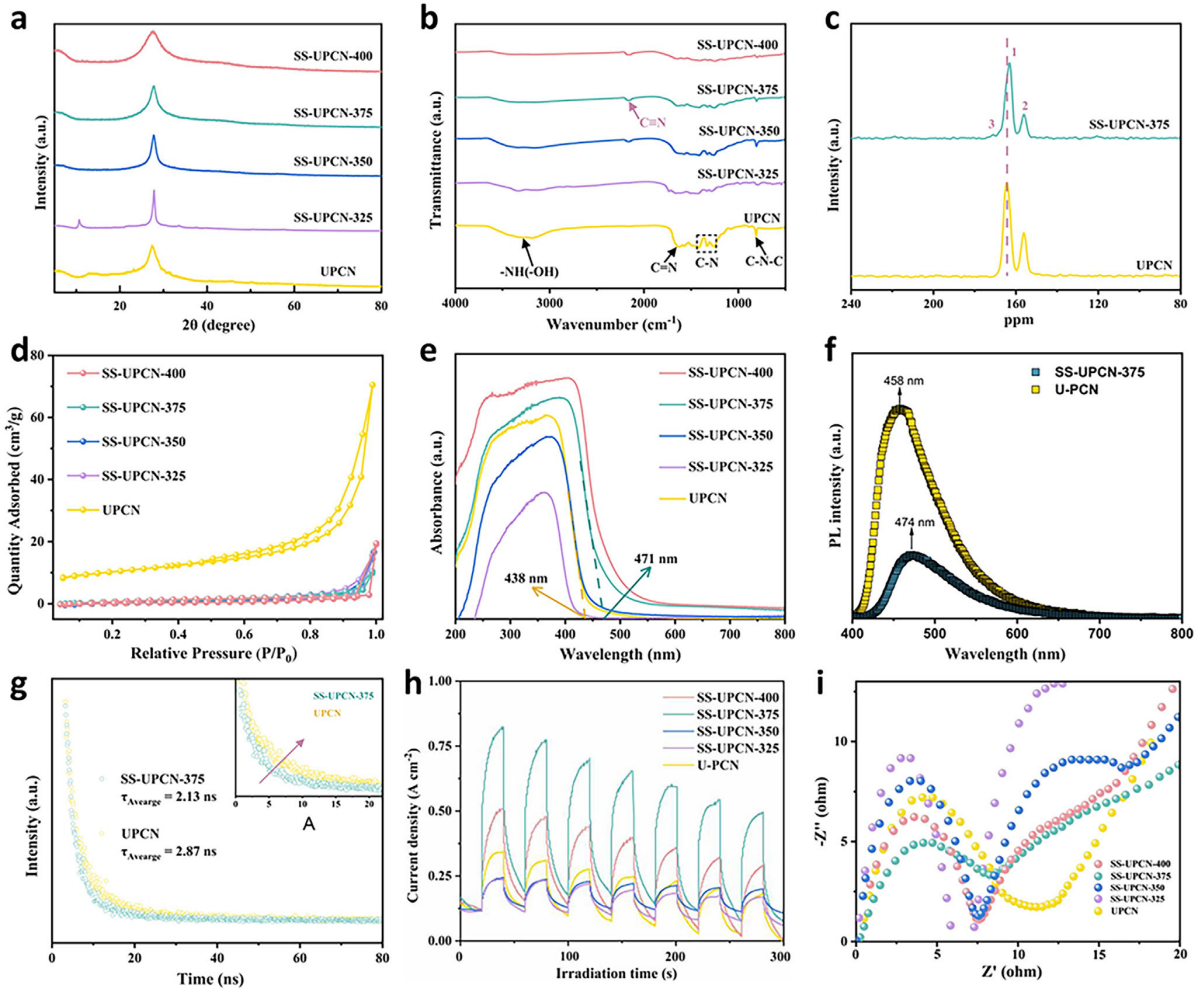
Phase composition and photoelectronic properties: a) XRD patterns, b) FT‐IR spectra, c) ^13^C NMR spectra, d) N_2_ adsorption–desorption isotherms, e) UV–Vis DRS spectra, f) steady‐state PL spectra, g) TR‐PL spectra, h) transient photocurrent response curves and i) EIS Nyquist plots of UPCN and SS‐UPCN‐T samples.

The chemical structure of all the SS‐UPCN‐T samples was characterized via XRD, FT‐IR, solid‐state ^13^C NMR and XPS spectroscopy. As shown in **Figure** **2**a, the synthesis of pristine UPCN through traditional high‐temperature (550 °C) thermal polymerization yielded two distinct diffraction peaks at 27.4° and 12.8°. These peaks corresponded to the (002) and (100) crystal planes, respectively, indicative of interlayer stacking in the conjugated aromatic system and repetitive units in the continuous heptazine framework, respectively.^[^ ^23^ ^]^ Remarkably, the characteristic diffraction peaks of the SS‐UPCN‐T, prepared according to the solar salt method, showed significant changes. As shown in Figure 2a, for SS‐UPCN‐325, the diffraction peak corresponding to the (100) crystal plane shifts from 12.8° (in UPCN) to 10.6°, indicating an expansion of the in‐plane repeating unit structure due to the insertion of alkali ions.^[^ ^24^ ^]^ With increasing thermal‐treatment temperature, the diffraction peak at 10.6° gradually disappears, which is attributed to localized structural disordering caused by the partial transformation of heptazine units to triazine configurations within the SS‐UPCN‐T structure under high‐temperature conditions.^[^ ^16^, ^18^, ^25^ ^]^ Additionally, the characteristic diffraction peak of the crystal plane shifted from 27.4° in UPCN to 27.8° in SS‐UPCN‐375 and SS‐UPCN‐400, marking a decrease in the interlayer distance from 0.328 to 0.321 nm. This distance reduction enhanced interlayer interactions, which facilitated carrier polarization and rapid charge migration between adjacent layers.^[^ ^26^ ^]^ Moreover, the observed sharpening and narrowing of the (002) crystal plane's characteristic diffraction peak also suggested enhanced crystallinity. In contrast, the samples prepared with a single salt (UPCN‐NaNO_3_‐375 and UPCN‐KNO_3_‐375) did not present the two characteristic peaks at 27.8 and 10.6° but showed additional diffraction peaks at 19.8, 21.5, 24.7, 33.8 and 37.6° (Figure S3, Supporting Information). These additional peaks indicated incomplete polymerization of the urea precursor under single‐salt conditions and low temperatures, as evidenced by the XRD characteristic peaks of the UPCN‐375. The results decisively demonstrated that the direct mixing of urea monomers with solar salt enabled the low‐temperature synthesis of SS‐UPCN‐T. The main reason for the notable discrepancy between XRD and HR‐TEM results may cause by that the solar salt promoted the rapid and orderly stacking of PCN on the *x* and *y* crystal planes at high temperature, whereas the stacking while on the *z* plane was relatively slow.^[^ ^16^ ^]^

As depicted in Figure 2b, FT‐IR spectra were employed to analyze the chemical structure of the synthesized catalysts. Two characteristic peaks at 807 cm^−1^ and within 1200–1700 cm^−1^ range can be found, which were attributed to the breathing and stretching modes of triazine or heptazine C─N rings, respectively.^[^ ^27^ ^]^ Furthermore, the UPCN samples exhibited pronounced absorbance bands within the 3100–3500 cm^−1^ range, which were indicative of the stretching modes of surface ─NH/─OH groups. This characteristic suggested a relatively lower degree of polymerization in the UPCN structure. In comparison, the SS‐UPCN‐T samples exhibited a consistent decrease in the FT‐IR signal intensity between 3000 and 3500 cm^−1^ as the heat treatment temperature increased, which was due to the enhanced polymerization processes facilitated by solar salts. A new absorption band at 2180 cm^−1^ was identified in the SS‐UPCN‐350, SS‐UPCN‐375 and SS‐UPCN‐400 samples, it mainly assigned to characteristic of the asymmetric stretching vibration of the cyano group (C≡N), and it can be attributed to the interaction between alkali ions and the cyano groups on urea‐derived intermediates during the thermal polymerization processes.^[^ ^24^ ^]^ Furthermore, solid‐state ^13^C MAS NMR spectra were employed to elucidate the structural alterations resulting from solar salt treatment compared with UPCN counterpart. As illustrated in Figure 2c, UPCN featured two prominent resonance peaks, centered at 163.1 and 155.6 ppm, which are corresponded to the (NH)─CN_2_ structure (peak 1) and C─N_3_ units (peak 2), respectively, and they can signify the presence of distinctive heptazine‐based structures within the UPCN.^[^ ^17^, ^18^ ^]^ However, a significant shift to lower values, specifically to 162.3 and 154.2 ppm, was observed in SS‐UPCN‐375 and SS‐UPCN‐400, which can be attributed to the intercalation of alkali ions within the C─N structural framework.^[^ ^26^, ^28^ ^]^ In addition, peak 3 at 171.6 ppm may attribute to (N─)─CN_2_ units, which have been caused by the negatively charged groups evidently.

XPS technology was employed to confirm the surface elemental compositions of UPCN and SS‐UPCN‐T samples. The XPS survey spectrum of SS‐UPCN‐T revealed the presence of C, N, O, Na, and K elements, whereas the spectrum for UPCN displayed only C, N, and O, which indicated the incorporation of K^+^ and Na^+^ into SS‐UPCN‐T (Figure S4a, Supporting Information). The chemical states were further confirmed through high‐resolution XPS spectra. As depicted in Figure S4b (Supporting Information), the C 1s spectra of UPCN and SS‐UPCN‐T displayed distinct peaks at binding energy of 288.0 and 284.6 eV, respectively, which corresponded to the sp^2^ hybridized carbon (N−C═N) in the UPCN and the surface‐contaminated carbon (C−C).^[^ ^29^ ^]^ Additionally, distinct peaks at 286.3, 292.7, and 295.4 eV were observed in SS‐UPCN‐T samples, which corresponded to the cyanic group (−C≡N), K 2p_3/2_ and K 2p_1/2_, respectively.^[^ ^18^ ^]^ Notably, an increase in the thermal treatment temperature resulted in a continuous rise in the −C≡N group content, which suggested that the reaction between solar salt and urea monomers intensified with increasing temperatures. These spectral features agreed with the findings from the FT‐IR spectra and ^13^C NMR analysis. As illustrated in Figure S4c (Supporting Information), N 1s XPS spectra of UPCN and SS‐UPCN‐T presented three distinct peaks at binding energy of 398.4, 399.5, and 400.8 eV, which represented bi‐coordinated N (C─N═C, N_2_C), tri‐coordinated N (N─(C)_3_, NC_3_) and NH_X_ groups, respectively.^[^ ^30^ ^]^ More importantly, the N 1s shifted toward the low binding energy in SS‐UPCN‐T, which indicated that the alkali ions had a strong interaction with the N 1s species.

The elemental composition and structural characteristics were examined through elemental analysis, ICP, and BET techniques. As indicated in Table S1 (Supporting Information), the C/N molar ratios of the SS‐UPCN‐T samples remained relatively stable with the increase in thermal treatment temperature, which suggested that the incorporated Na^+^ and K^+^ ions primarily occupied interstitial positions in the UPCN structure, without substituting C or N atoms. Furthermore, the ICP results demonstrated a plateau in the Na^+^ and K^+^ content with the elevation of the thermal treatment temperature after 375 °C. Additionally, N_2_ adsorption–desorption experiments disclosed that SS‐UPCN‐T exhibited a notable reduction in specific surface area and pore volume compared with UPCN (Table S1, Supporting Information and Figure 2d), further verifying the higher crystallinity of SS‐UPCN‐T samples produced from low‐temperature solar salt approach.

UV–vis DRS were employed to evaluate the photoelectronic properties on the effect of the solar salt‐assisted polymerization for the photocatalysts. As shown in Figure 2e, with an increase in thermal treatment temperature, a marked enhancement in optical absorption within the π–π* transition regions were observed for SS‐UPCN‐T samples. This enhancement also resulted in a distinct color transition from pale to bright yellow. Notably, for SS‐UPCN‐375, a significant redshift in the optical absorption edge was observed, shifting from 438 nm to around 471 nm. The Tauc plot results revealed that the bandgap of SS‐UPCN‐375 photocatalyst (2.74 eV) was narrower than that of UPCN (2.94 eV), and implying enhanced optical utilization upon electron transition processes (Figure S5a, Supporting Information). As detailed in Figure S5b (Supporting Information), VB‐XPS spectra was used to further estimate the valence band edge positions (*E*_VB_) of the synthesized samples. The *E*_VB_ values for all samples were found to be nearly identical, and the value was approximately 1.70 eV. Utilizing the equation of *E*_CB_ = *E*_VB_ − *E*_g_, the conduction band (*E*_CB_) positions of SS‐UPCN‐375 and UPCN were determined to be −1.24 and −1.04 eV versus normal hydrogen electrode as shown in Table S2 (Supporting Information). In comparison to UPCN, although the *E*_CB_ of SS‐UPCN‐375 was shifted toward more positive potentials, the band structure of SS‐UPCN‐375 was distinctly modulated, enhancing visible light absorption and superior redox capabilities. The changes in the band arrangement are expected to provide a substantial thermodynamic driving force for the oxygen reduction reaction (ORR).

The influence of solar salt‐assisted synthesis on SS‐UPCN‐T structures and their impact on photocatalytic redox reactions were further investigated by evaluating the efficiency of separated and migrated photogenerated carriers. As shown in Figure 2f, UPCN exhibited a prominent and broad emission peak centered at 458 under 325 nm excitation. In contrast, SS‐UPCN‐375 showed a weaker and red‐shifted emission peak centered around 474 nm, suggesting suppressed radiative recombination of photoexcited charge carriers.^[^ ^31^ ^]^ Furthermore, the average radiative lifetimes of charge carriers for UPCN and SS‐UPCN‐375 were determined to be 2.87 and 2.13 ns by fitting double‐exponential functions to their respective time‐resolved photoluminescence (TR‐PL) peaks (Figure 2g). The average fluorescence decay lifetime in SS‐UPCN‐375 was significantly shorter than that of UPCN, suggesting an enhanced dissociation of excitons, primarily because of the decreased interlayer spacing and increased crystallinity in SS‐UPCN‐375.^[^ ^17^, ^32^ ^]^ These structural features are conducive to expediting the charge transfer from the interlayers to the interface or surface, thereby minimizing recombination. As shown in Figure 2h,i, with the increase of sample preparation temperature, the photocurrent intensity increases first and then decreases. On the contrary, the impedance intensity decreases first and then increases with the increase of sample preparation temperature. When the preparation temperature is 375 °C, SS‐UPCN‐375 has the strongest photocurrent intensity and the weakest impedance intensity, which indicates that it has the best photogenerated carrier separation and migration performance. Furthermore, these results were also substantiated by the electron paramagnetic resonance (EPR) test on UPCN and SS‐UPCN‐375 (Figure S6, Supporting Information). SS‐UPCN‐375 had a higher number of unpaired electrons within its aromatic system compared with UPCN. Although the *g*‐value remained unchanged, a marked increase in signal intensity was noted for SS‐UPCN‐375, indicating the presence of numerous free charge carriers in the localized heterocyclic ring.^[^ ^33^ ^]^

### 2.3 Exciton Dissociation and Carrier Dynamics Study

**Figure 3:**
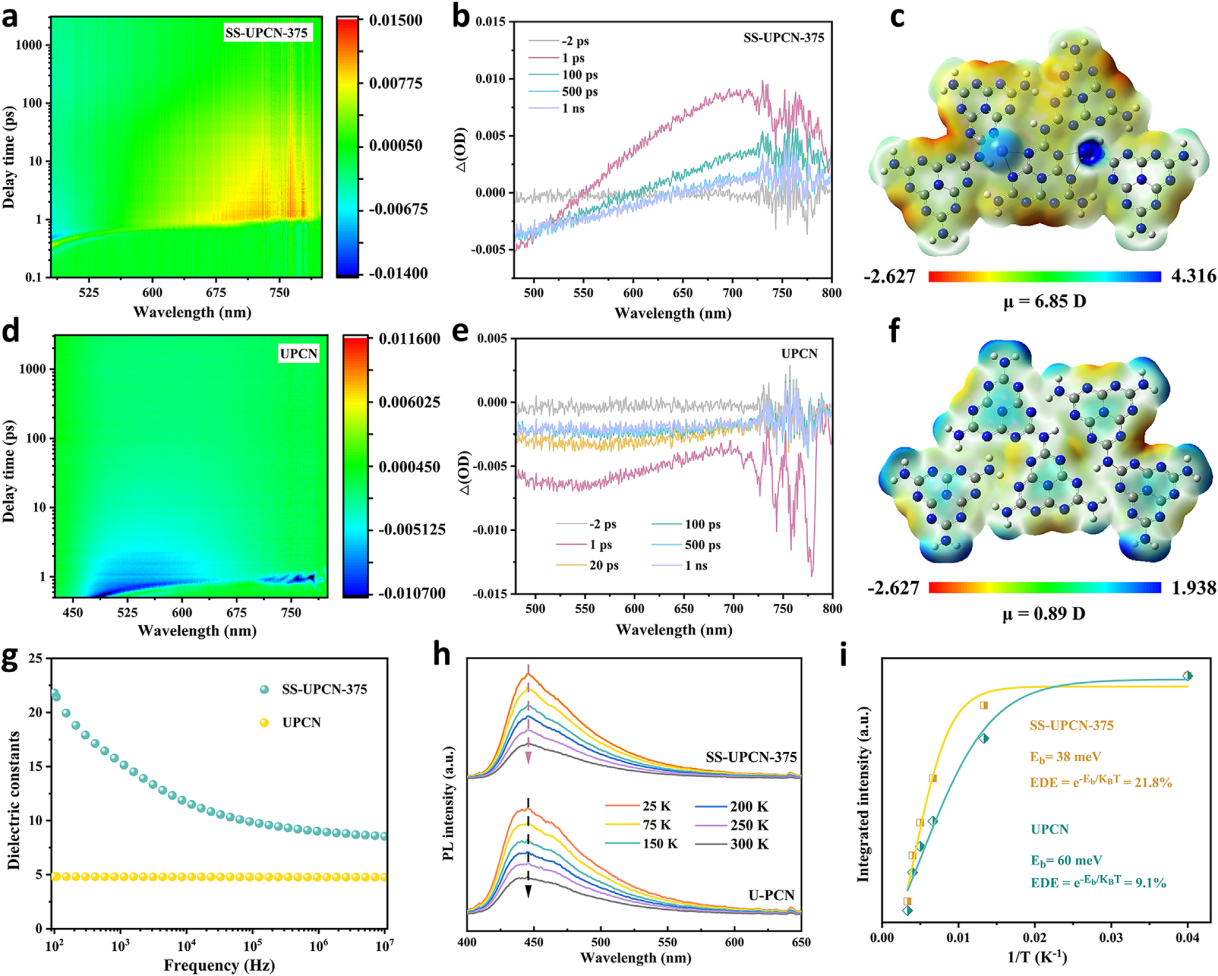
Time‐wavelength‐dependent TA color maps, TA spectra at different probe delays and decay kinetics probed at 700 nm, illustration depicting the distribution of electron and molecular dipoles on a–c) SS‐UPCN‐375 and d–f) UPCN, g) dielectric constant of UPCN and SS‐UPCN‐375, h) temperature‐dependent PL and i) integrated PL intensity as a function of temperature from 25 to 300 K.

Femtosecond ultrafast transient absorption (fs‐TAS) is a crucial technique used to track relaxation pathways and study the dynamics of photoinduced carriers.^[^ ^34^ ^]^ The UPCN and SS‐UPCN‐375 samples were excited using a 360 nm pump laser, transitioning them from ground state to an excited state. **Figure** **3**a,d displays the fs‐TA spectra (450–800 nm) at various probe delays, illustrating the signals of ground‐state bleaching (GSB) and excited‐state absorption (ESA). Compared with UPCN, SS‐UPCN‐375 exhibited the most robust and prolonged absorption for ESA, indicating that its structure, synthesized with the aid of solar salt, effectively promoted the accumulation of shallowly trapped electrons within the system. To further elucidate the relationship between shallow and deep trapping states, the decay kinetics of UPCN and SS‐UPCN‐375 at 700 nm over 1000 ps using a double‐exponential function were analyzed. The τ_1_ value represented the trapping of shallow electrons, and the longer‐lived time constant τ_2_ was associated with the less favorable deep trapping states, which deactivated the free electrons and holes, leading to decreased photocatalytic activities. As presented in Figure 3b,e, the kinetic fitting results revealed two‐time constants: *τ*_1_ = 81.23 fs and *τ*_2_ = 244 ps for UPCN, and *τ*_1_ = 3.2 ps and *τ*_2_ = 100.5 ps for SS‐UPCN‐375, respectively. The increased *τ*_1_ component in SS‐UPCN‐375 (60.4 vs 11.8%) suggested that more excitons were trapped by shallow traps and subsequently dissociated into free carriers, whereas the decreased *τ*_2_ component (39.6 vs 88.2%) indicated fewer excitons being trapped and accumulated by deep traps.^[^ ^35^ ^]^

As shown in Figure 3c,f, the electronic structure of SS‐UPCN‐T samples experienced notable alterations after low‐temperature solar salt‐thermal treatment. Significantly, the molecular dipole moments of SS‐UPCN‐375 surged from 0.89 to 6.85 Debye, marking a considerable enhancement over UPCN counterpart. This elevation was crucial for facilitating the separation of electron–hole pairs, thus enhancing the efficiency of the photocatalytic reaction.^[^ ^36^ ^]^ Concurrently, these enhanced local dipole moments, which contribute to generating a polarized electric field, resulting in a marked macroscopic polarization effect.^[^ ^37^ ^]^ This promotion effect essentially originates from the enlargement of the dielectric constant (*ε*_r_), as demonstrated in Figure 3g. The dielectric constants of both UPCN and SS‐UPCN‐375 were determined through the impedance analysis were conducted on the capacitors. UPCN displayed a comparatively low *ε*_r_, spanning from 5 to 7 across the analyzed frequency spectrum. SS‐UPCN‐375 displayed a dramatically rising *ε*_r_ value as frequency decreased, reaching values exceeding 20 in the low‐frequency range. This increased dielectric constant aided in promoting the effective separation of tightly bound Frenkel excitons in the SS‐UPCN‐375.

As shown in Figure 3h, temperature‐dependent photoluminescence (TD‐PL) spectra were performed to determine the exciton binding energies (*E*_b_) of UPCN and SS‐UPCN‐375 samples. A notable decrease in the intensity of the PL peak was found as the temperature increased gradually from 25 to 300 K. The E_b_ values were determined by the Arrhenius equation: $I\;\left(T\right)=I_{0}\;/\left(1+Ae^{-\frac{E_{b}}{K_{B}T}}\right)$, where *I*_0_ is the PL intensity at 0 K, *K*_B_ is the Boltzmann constant, and *E*_b_ is the binding energy of exciton. It indicated that the *E*_b_ for SS‐UPCN‐375 was determined to be 38 meV, which was markedly lower than the value of 60 meV recorded for UPCN (Figure 3i). Moreover, the exciton dissociation efficiency in SS‐UPCN‐375, quantified at 21.8% according to the equation $\mathrm{EDE}=e^{-E_{b}/\left(K_{B}T\right)}$, where *k*_B_ *T* ≈ 25 meV at room temperature, was found to be 2‐times higher than that of UPCN (9.1%). Relative to UPCN, the ameliorative *E*_b_ and EDE of SS‐UPCN‐375 implied that notably promotion of charge transport and collection capabilities within its structure, which was conducive to the migration of free electrons and holes to the catalyst surface, thus heightening H_2_O_2_ photocatalytic performance.

**Figure 4:**
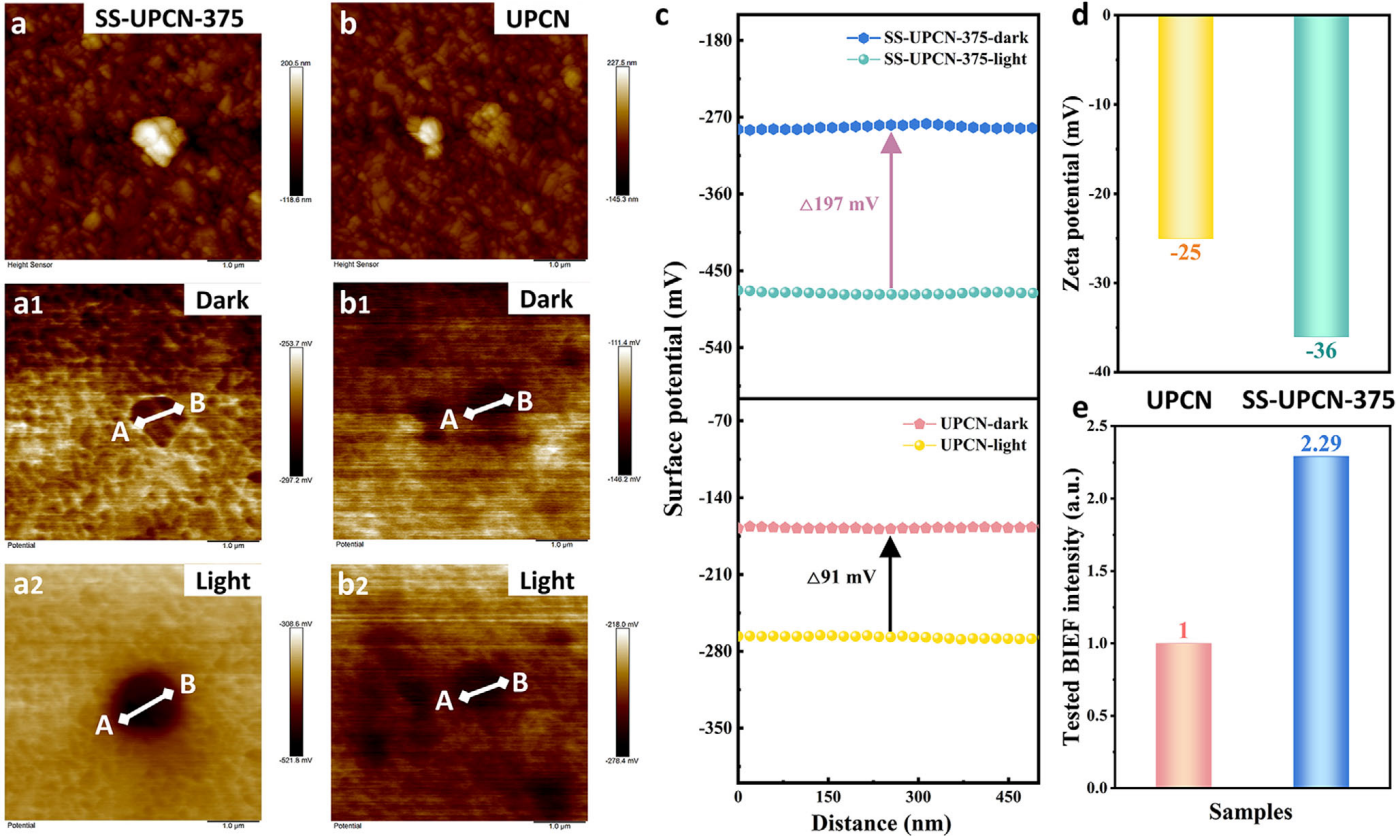
a,b) Surface morphology and corresponding surface potential images in a1,b1) the absence and a2,b2) presence of light for UPCN and SS‐UPCN‐375 samples, c) surface potential difference profiles across UPCN and SS‐UPCN‐375 before and after irradiation, d) Zeta potential values, and e) tested BIEF intensity (assuming the intensity of UPCN before and after light irradiation to be “1”) of SS‐UPCN‐375 sample.

To delve deeper into the surface potential of the UPCN and SS‐UPCN‐T samples, Kelvin probe force microscopy (KPFM) was employed. As illustrated in **Figure** **4**a,b, the surface potential images showed a negative potential variation for both UPCN and SS‐UPCN‐375 within the scanning window, signifying that the primary form of surface photogenerated charge mainly consisted of photogenerated electrons.^[^ ^38^, ^39^ ^]^ The contact potential difference (ΔCPD) was closely related to the surface potential. As depicted in Figure 4a1,a2, upon illumination, the charge separation resulted in an augmented interlayer charge accumulation in the negative direction. Figure 4c illustrates the difference in ΔCPD across different photocatalysts, showing an increase from 91 mV for UPCN to 197 mV for SS‐UPCN‐375. The larger ΔCPD observed in SS‐UPCN‐375 suggested an accelerated accumulation of electrons and holes in the surface and bulk regions, respectively. This phenomenon was attributed to the enhanced crystallinity of SS‐UPCN‐375, which generated a larger internal built‐in electric field (BIEF).^[^ ^13^ ^]^ The most intensive ΔCPD signal on SS‐UPCN‐375 reflected the boosted separation of holes and electrons, which is believed to be attributed to the driving force via the stronger BIEF. Additionally, the increased BIEF intensity before and after light irradiation was further estimated by using the model developed by Kanata, in which the BIEF intensity was determined by the surface potential and surface charge density (Equation S1 in Supporting Information). The zeta potentials of UPCN and SS‐UPCN‐375 were ‐25 and −36 mV, respectively (Figure 4d), and the corresponding surface charge density was measured by using Gouy‐Chapman (Equations S2 and S3 in Supporting Information). Finally, the calculated BIEF intensity before and after light irradiation of HTCN was 2.29, which was 2.29 times stronger than UPCN (Figure 4e). Accordingly, the successful construction of crystalline PCN via one‐pot solar salt thermal polymerization effectively boosted the superior charge separation via the increased BIEF intensity and then facilitated its photocatalytic activities.

### 2.4 The Proposed H_2_O_2_ Photosynthesis Mechanism

**Figure 5:**
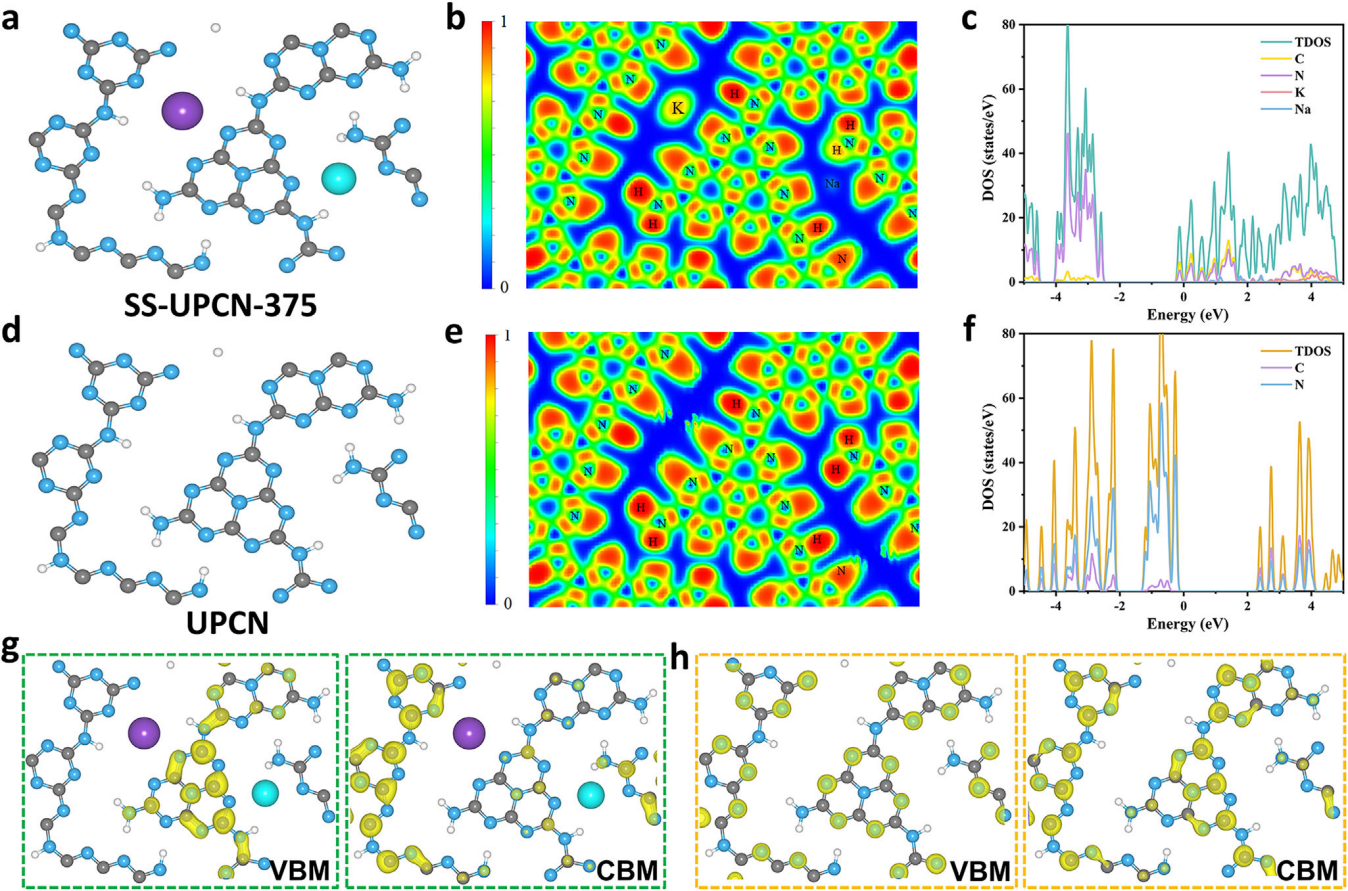
The optimized structures, electronic localization function, DOS calculations and VBM and CBM states of a–c,g) SS‐UPCN‐375 and d–f,h) PCN samples. The atoms of C, N, K, Na and H are in gray, blue, purple, cyan and white, respectively.

As depicted in **Figure** **5**a, the presence of trace amounts of Na^+^ and K^+^ in the sample was confirmed to induce alterations in electronic localization and charge carrier mobility. The electronic structures of UPCN and SS‐UPCN‐375 were investigated using the plane‐wave technique, as implemented in the Vienna ab initio Simulation Package.

Compared with the electronic structure of pristine UPCN, the insertion of Na^+^ and K^+^ into amine‐linked heptazine‐based melon chains resulted in a significant increase in electron densities (Figure 5a–e) in the SS‐UPCN‐375 structure. In addition, the calculated densities of states (DOS, Figure 5c,f) exhibited typical semiconductor properties, with the Fermi level positioned at 0 eV between the valence band maximum (VBM) and conduction band minimum (CBM). Electrons originating from alkali metal ions partially occupied the CB, which led to a reduction in the CB position and consequently a narrowing of the bandgap, which was consistent with experimental findings. Figure 5g,h indicated the band‐decomposed charge densities of VBM and CBM. Compared with the symmetric distribution in UPCN, the distribution in SS‐UPCN‐375 was more asymmetric and localized. Because of the dramatically increased electron densities in melon chains around alkali metal ions, VBM became localized in the region far from Na^+^ and K^+^. This spatial separation of VBM and CBM states facilitated the efficient separation of photo‐generated carriers.

**Figure 6:**
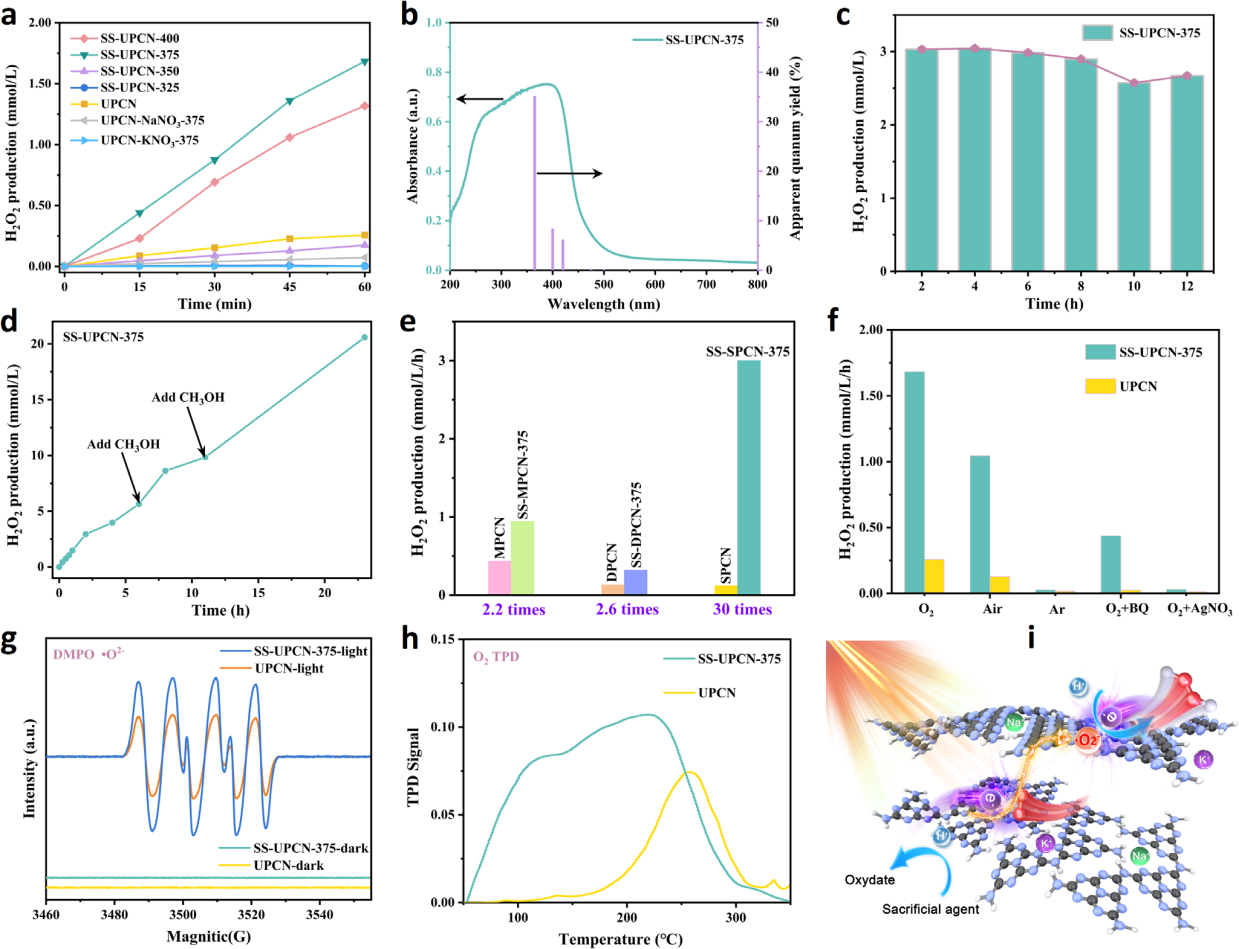
The photocatalysis performance and proposed mechanism: a) H_2_O_2_ production performance on UPCN and SS‐UPCN‐T samples, b) AQY of H_2_O_2_ produced on SS‐UPCN‐375 sample, c) cycling stability of SS‐UPCN‐375, d) H_2_O_2_ production on SS‐UPCN‐375 for a long‐term run, e) H_2_O_2_ production performance of PCN prepared by different precursors, f) H_2_O_2_ production performance of UPCN and SS‐UPCN‐375 samples under different reaction atmospheres and external sacrificial agents, g) ESR spectra, h) O_2_ TPD, and i) proposed mechanism on SS‐UPCN‐375.

Employing low‐temperature solar salt‐assisted thermal treatment to construct optimized PCN structure with high crystallinity and enhanced carrier mobility significantly enhanced its photocatalytic selective oxygen reduction (ORR) performance. Among them, H_2_O_2_ photosynthesis by pristine UPCN and SS‐UPCN‐T photocatalysts was evaluate. As depicted in **Figure** **6**a, the pristine UPCN photocatalyst without solar salt treatment exhibited relatively low photocatalytic activity, which achieved only 0.27 mmol L^−1^ h^−1^ H_2_O_2_ production. Conversely, with solar salt‐assisted low‐temperature thermal treatment, the photocatalytic H_2_O_2_ production activity of SS‐UPCN‐T photocatalyst was obviously promoted. The highest activity was observed at a thermal treatment temperature of 375 °C, where the SS‐UPCN‐375 photocatalyst achieved an H_2_O_2_ production activity of 1.80 mmol L^−1^ h^−1^, which was approximately 6.7 times higher than that of UPCN. It was confirmed that constructing localized crystalline structures under these synthetic conditions significantly accelerated exciton dissociation and charge carrier transfer, thereby enhancing photocatalytic activity. The H_2_O_2_ evolution rates of the SS‐UPCN‐375 photocatalyst are more efficient among the recently reported photocatalytic reaction systems (Table S3, Supporting Information). Notably, the H_2_O_2_ production activity on samples obtained from single‐component salt NaNO_3_ (UPCN‐NaNO_3_‐375, 0.07 mmol L^−1^ h^−1^) or KNO_3_ (UPCN‐KNO_3_‐375, 0.001 mmol L^−1^ h^−1^) displayed inferior photocatalytic activity, further verifying that single salt component did not facilitate performance improvement.

Moreover, the apparent quantum yield (AQY) of SS‐UPCN‐375 and UPCN for photocatalytic H_2_O_2_ production at different wavelength (*λ*  = 365, 400, and 420) were measured and shown in Figure 6b and Figure S7 (Supporting Information), respectively. It is evident that the SS‐UPCN‐375 showed higher AQY (i.e., 35.0, 8.2, and 6.0% at 365, 400, and 420 nm, respectively), whereas UPCN showed lower AQY (i.e., 2.6, 1.7, and 0.05% at 365, 400, and 420 nm, respectively). This wavelength‐dependent AQY trend corresponded well with the absorption spectrum of SS‐UPCN‐375, indicating that the H_2_O_2_ generation was a photocatalytic process. Besides, the H_2_O_2_ production performance of SS‐UPCN‐375 at different catalyst concentrations was assessed and shown in Figure S8a (Supporting Information). As the concentration of SS‐UPCN‐375 increased from 0.2 to 4.0 g L^−1^, the H_2_O_2_ production rate gradually increased and subsequently decreased. The maximum photocatalytic activity for H_2_O_2_ production reached 1.80 mmol L^−1^ h^−1^, which was achieved on SS‐UPCN‐375 with the concentration of 2.0 g L^−1^. Further increase in the concentration of SS‐UPCN‐375 to 4.0 g L^−1^ led to a slight decrease in activity. This decrease were attributed to the light‐shielding effect resulting from excessively high catalyst concentrations. In addition, the H_2_O_2_ yields using SS‐UPCN‐375 with various mass ratios of solar salt to urea (m_solar salt_/_urea_) were also examined and demonstrated in Figure S8b (Supporting Information). With the m_solar salt/urea_ increasing from 10 to 25 wt%, the H_2_O_2_ production rate firstly increased and then decreased, and finally reached an optimal value when the m_solar salt/urea_ amount was 20 wt%. As shown in Figure S9 (Supporting Information), under the optimal catalyst preparation conditions, the activity of the photocatalyst was still greatly improved by using other binary and ternary solar salts, and was much higher than that of the catalyst treated with Li salt, which indicated that the method has certain universality. Figure 6c shows the cycling stability of the SS‐UPCN‐375 photocatalyst under visible light, and only a slight decrease in activity after six cycles (12 h), which could be attributed to the inadequate of sacrificial agent. Furthermore, during prolonged H_2_O_2_ production using SS‐UPCN‐375, a decline in the H_2_O_2_ production rate occurred when the accumulated H_2_O_2_ amount was high (about 5 mm). This phenomenon was likely due to the consumption of the methanol sacrificial agent. As depicted in Figure 6d, following the replenishment of methanol, a sustainable growth of the H_2_O_2_ production rate was further observed. These results strongly indicated that the SS‐UPCN‐375 photocatalyst exhibited robust photocatalytic stability for H_2_O_2_ photosynthesis.

Additionally, the H_2_O_2_ production performance of UPCN and SS‐UPCN‐375, which were synthesized by different nitrogen‐rich precursors (e.g., melamine, dicyandiamide, and thiourea), were also assessed (Figure 6e). It was found that the H_2_O_2_ production activities by different precursors were also significantly enhanced, indicating the universality of the low‐temperature solar salt‐assisted thermal treatment method. In order to determine the active sites and analyze the formation mechanism of H_2_O_2_, DFT theoretical calculations and in situ infrared spectroscopy were used. As shown in Figure S10 (Supporting Information), the adsorption behavior of O_2_ on PCN and SS‐UPCN‐375 catalysts was systematically studied. The results show that the adsorption energy of O_2_ at the N1 site is the lowest among the pure PCN catalysts, indicating that this site is the most stable adsorption site. In contrast, the adsorption behavior of O_2_ changes significantly for SS‐UPCN‐375 catalysts, which original adsorption structure at the N1 site relaxes, and the adsorption center shifts to the vicinity of the alkali metal ion (K^+^ or Na^+^). Further comparison revealed that the adsorption energy of O_2_ at the K^+^ top site is the lowest, indicating that this site becomes the most energetically active adsorption center in the SS‐UPCN‐375 system. As shown in Figure S11 (Supporting Information), in situ infrared spectroscopy was further employed to analyze the intermediate species generated during the reaction. The bands at 1134 cm^−1^ and 1209 cm^−1^ were assigned to the O─O stretching mode of surface‐adsorbed •O_2_^−^ and surface‐adsorbed superoxide (*OOH), respectively.^[^ ^40^, ^41^ ^]^ The peaks observed at 1406–1442 cm^−1^ and 1491 cm^−1^ were attributed to the OOH bending mode of surface‐adsorbed hydroperoxide (*H_2_O_2_) and adsorbed molecular oxygen (*O_2_).^[^ ^42^, ^43^ ^]^ Notably, with prolonged irradiation time, the O─O stretching vibrations of surface‐adsorbed *O_2_, O_2_^−^, and *OOH, as well as the OOH bending mode of surface‐adsorbed *H_2_O_2_ gradually intensified, implying the sustained formation of the aforementioned reactive oxygen species (ROS) intermediates. To better elucidate the detailed pathways of H_2_O_2_ formation and understand the O_2_ ORR activation mechanism, several sacrificial agents control experiments were conducted. Silver nitrate (AgNO_3_) and benzoquinone (BQ) were used as electron (e^−^) and superoxide radical (•O_2_^−^) scavengers, respectively. As illustrated in Figure 6f, when air was used instead of O_2_, the H_2_O_2_ yield decreased to only 58%. Furthermore, when Ar was used as a replacement for O_2_, only trance H_2_O_2_ was detected. This result indicated that O_2_ was an essential reactant for photocatalytic H_2_O_2_ production and that the O_2_ reduction pathway was the primary route for H_2_O_2_ photosynthesis in this study. When •O_2_^−^ was eliminated by BQ, a significant reduction in H_2_O_2_ production, providing additional confirmation that the two‐step single‐electron ORR pathway was responsible for H_2_O_2_ generation, and •O_2_^−^ served as a crucial intermediate in the artificial photosynthesis of H_2_O_2_. Furthermore, when an electron scavenger (AgNO_3_) was introduced into the reaction system, H_2_O_2_ was hardly detectable, further confirming that the photocatalytic generation of H_2_O_2_ primarily occurred through ORR. The ESR test conducted using 5,5‐dimethyl‐1‐pyrroline N‐oxide (DMPO) as a •O_2_^−^ capture agent revealed a significantly higher •O_2_^−^ signal on SS‐UPCN‐375 under visible light irradiation compared with that on UPCN (Figure 6g). Combining insight into the oxygen temperature‐programmed desorption (O_2_‐TPD) analysis, it was evident that SS‐UPCN‐375 exhibited a markedly stronger O_2_ adsorption capacity in comparison to UPCN (Figure 6h). The O_2_ molecules adsorbed on SS‐UPCN‐375 could undergo redox reactions with photogenerated carriers that rapidly migrated to the surface, resulting in the generation of H_2_O_2_. This result further substantiated the involvement of the two‐step single‐electron ORR pathways during the H_2_O_2_ generation processes (O_2_ + e^−^ → •O_2_^−^; •O_2_^−^ + H^+^ + e^−^ →•OOH; •OOH + H^+^→H_2_O_2_), the proposed mechanism was finally illustrated in Figure 6i.

## 3 Conclusions

In summary, this study innovatively utilizes the efficient thermal storage of solar salt to rapidly induce low‐temperature crystallization of heptazine‐based melon, culminating in the creation of a PCN photocatalyst with enhanced crystallinity and superior exciton dissociation capability. Furthermore, comprehensive characterizations coupled with DFT calculations were applied to reveal the exceptional photocatalytic performance of H_2_O_2_ photosynthesis originated from the aspects of meticulously engineered structure, significantly enhanced optical absorption, and refined photoelectronic properties on SS‐UPCN‐375 photocatalyst. Overall, a simple, ecofriendly, and economical approach for the cost‐effective production of high‐performance PCN‐based photocatalysts for energy and environmental‐related applications was well‐established and implemented in this paper.

## 4 Experimental Section

### Preparation of Photocatalysts

In this study, four kinds precursors (urea, melamine, dicyandiamide, and thiourea) and binary mixed solar salt (60% NaNO_3_–40% KNO_3_) were used for PCN synthesis. The final obtained product was denoted as SS‐XPCN‐T (where X represents the precursor and T represents the calcination temperature). Specifically, as illustrated in Figure 1a, 10 g of solar salt and specific amounts of the precursors were meticulously mixed in an agate mortar. The resultant mixtures were then placed in a covered alumina crucible and thermally heated in an oven to 300–400 °C at a rate of 2 °C min^−1^, maintaining this temperature for an additional 2 h. Subsequently, it allowed the temperature to cool naturally to room temperature before collecting the final products. Following an extensive washing with boiling water to eliminate any remaining solar salt, the products were then subjected to drying in a vacuum oven at a temperature of 80 °C for a duration of 24 h to yield a deep‐yellow powder. For simplicity, samples were donated synthesized with 60% NaNO_3_‐40% KNO_3_ solar salt using various monomers and temperatures as SS‐UPCN‐T (urea), SS‐MPCN‐T (melamine), SS‐DPCN‐T (dicyandiamide), and SS‐SPCN‐T (thiourea), respectively. For comparative purposes, samples were labeled synthesized without solar salt or with individual salts, specifically NaNO_3_ or KNO_3_, as UPCN‐T, MPCN‐T, DPCN‐T, SPCN‐T, UPCN‐NaNO_3_‐T, and UPCN‐KNO_3_‐T, respectively. UPCN was synthesized following the method outlined in previous report.^[^ ^44^ ^]^ Typically, 10 g of each monomer was placed in a covered crucible and subjected to polymerization in a muffle furnace at 550 °C for 2 h.

### Photocatalytic H_2_O_2_ Production Evaluation

The concentration of H_2_O_2_ in the experiment was determined using the colorimetric method with PACKTEST (WAK‐H_2_O_2_, Kyoritsu Chemical‐Check Laboratory, Corp.), coupled with a digital PACKTEST spectrometer (ED723, GL Sciences Inc.).^[^ ^45^ ^]^ Typically, 100 mg of prepared photocatalyst was dispersed in 50 mL aqueous solution containing 10 vol% methanol in a quartz glass bottle (maximum diameter, φ 50 mm; capacity 250 mL). The solution underwent sonication for 10 min to ensure even dispersion of the photocatalyst, then followed by oxygenating for 30 min in the dark to attain O_2_ saturation. Subsequently, the bottle was maintained at 25 ± 0.5 °C in an oil bath and irradiated with a 300 W Xe lamp (Perfectlight, Beijing, China) equipped with a 400 nm cutoff filter. After a specified duration, the quantifiable solution was sampled from the container and analyzed using the PACKTEST instrument.

## Conflict of Interest

The authors declare no conflict of interest.

## Supporting information



Supporting Information

## Data Availability

The data that support the findings of this study are available from the corresponding author upon reasonable request.
