# Supplementary material for: A Low‐Temperature Solar Salt Approach to Fabricate Crystalline Polymeric Carbon Nitride for H2O2 Efficient Photosynthesis
Source: Adv Sci (Weinh). 2025 Oct 13;13(2):e12549. doi: 10.1002/advs.202512549 (PMC12786306; doi:10.1002/advs.202512549)
Supplement: Supplementary file 1 — Supporting Information [file ADVS-13-e12549-s001.docx]

**Supporting Information**

**A Low-Temperature Solar Salt Approach to Fabricate Crystalline Polymeric Carbon Nitride for H_2_O_2_ Efficient Photosynthesis**

Salt-Mediated Structural Transformation in Carbon

Nitride:

Salt-Mediated Structural Transformation in Carbon

Nitride:

Salt-Mediated Structural Transformation in Carbon

Nitride:

Salt-Mediated Structural Transformation in Carbon

Nitride:

Salt-Mediated Structural Transformation in Carbon

Nitride:

Salt-Mediated Structural Transformation in Carbon

Nitride:

Salt-Mediated Structural Transformation in Carbon

Nitride:

Salt-Mediated Structural Transformation in Carbon

Nitride:

Jie Tang^a^, Junqing Li^b^, Lu Li^a^，Chengying Xu^a^, Hui Yang ^c, e*^, Chao Chen^b^, Weiqiang Hao^a^, Yi Yang^a^, Kelin He^b^, Linfu Xie^b^, Feng Tang^b^, Zimo Huang^d*^, Qitao Zhang^b*^

^a^Changzhou Vocational Institute of Engineering, Changzhou, 213000, China

^b^International Collaborative Laboratory of 2D Materials for Optoelectronics Science and Technology of Ministry of Education, Institute of Microscale Optoelectronics, Shenzhen University, Shenzhen, 518060, China

^c^College of Environmental Science and Engineering, Yangzhou University, Yangzhou, 225127, China

^d^School of Metallurgy and Environment, Central South University, Changsha, 410083, China

^e^School of Chemistry and Chemical Engineering, Yangzhou University, Yangzhou, 225002, China

*Corresponding authors

E-mail: huiyang5211@yzu.edu.cn (H. Yang); [zimo.huang@csu.edu.cn (Z](mailto:zimo.huang@csu.edu.cn%20(Z).Huang); qitao-zhang@szu.edu.cn (Q. Zhang)

Salt-Mediated Structural Transformation in Carbon

Nitride:

Salt-Mediated Structural Transformation in Carbon

Nitride:

Salt-Mediated Structural Transformation in Carbon

Nitride:

Salt-Mediated Structural Transformation in Carbon

Nitride:

Salt-Mediated Structural Transformation in Carbon

Nitride:

Salt-Mediated Structural Transformation in Carbon

Nitride:

Salt-Mediated Structural Transformation in Carbon

Nitride:

Salt-Mediated Structural Transformation in Carbon

Nitride:

**Supplementary Experimental Section**

**S1. Chemicals**

All chemicals used in this investigation were of analytical quality and were utilized without further purification. Benzoquinone (BQ), silver nitrate (AgNO_3_), melamine, urea, thiourea and dicyandiamide were acquired from Aldrich Industrial Inc., China. Solar salt (60% NaNO_3_-40% KNO_3_) was purchased from Shanghai Ruidong Chemical Group Co., Ltd.

**S2. Characterizations**

Powder X-ray diffraction (XRD) patterns were obtained using a Bruker D4 diffractometer (Cu Kα radiation, λ = 1.54056 Å). Transmission electron microscopy (TEM), high-resolution TEM (HR-TEM), high-angle annular dark-field TEM (HAADF-STEM), selected area electron diffraction (SAED), and energy-dispersive X-ray spectroscopy (EDX) analyses were conducted on a Titan Cubed Themis G2 300 electron microscope at an accelerating voltage of 300 kV. The analysis of scanning electron microscopy (SEM) was carried out on a HITACHI S4800, where the beam energy was adjusted to 5 kV. Fourier transform infrared (FT-IR) spectra were recorded using a NICOLET iS10 spectrometer within the spectral range of 400 to 4000 cm^-1^. Solid-state ^13^C nuclear magnetic resonance (NMR) spectral analysis was conducted using a Bruker AVANCE NEO 400 WB. X-ray photoelectron spectroscopy (XPS) and valence-band X-ray photoelectron spectroscopy (VB-XPS) measurements were conducted using an XPS apparatus (Escalab 250xi, America), and the binding energies were calibrated against contaminated carbon (C 1s = 284.6 eV). UV-3600 Shimadzu spectrophotometer was utilized for the determination of UV-vis spectra. The surface potential of the photocatalyst was measured using photo-irradiated Kelvin probe force microscopy (KPFM) on a Bruker ICON instrument. Femto-second transient absorption spectroscopy (fs-TAS) were determined on Helios (ultrafast systems) spectrometer. Electron spin resonance spectra of spin-trapped paramagnetic species utilizing 5,5-dimethyl-1-pyrroline N-oxide (DMPO) in a methanol solvent were recorded with an A300-10/12 spectrometric apparatus. The electrochemical workstation (CHI660D, Shanghai, Chenhua) was utilized to gather transient photocurrent responses (I-t) and electrochemical impedance spectra (EIS). Photoluminescence (PL), time-resolved photoluminescence (TR-PL) and temperature-dependent photoluminescence (TD-PL) spectra were carried out in a HORIBA Fluorolog-3 instrument.

**S3. Experimental support for the local BIEF of the photocatalysts**

The BIEF magnitude of the obtained PCN with different structures was calculated by using the following equation (S1) developed by Kanata et.al [1].

Fs = (−2V*sρεε0*)^1/2^ (S1)

Where Fs, V*s*, *ρ*, *ε*, and *ε0* stand for the internal electric field magnitude, the surface potential, the surface charge density, the low-frequency dielectric constant, and the vacuum dielectric constant, respectively. The above equation reveals that the BIEF magnitude is mainly determined by the surface potential and charge density. The value of Vs is obtained by KPFM. The *ρ* is tested by the zeta potential and then calculated using the model given by Gouy-Chapman (S2) [2]:


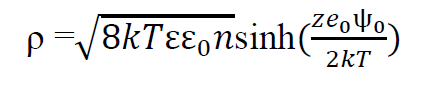
(S2)

Among them, for the smaller Zeta potential value (𝜓_0_ < 50 mV), they are approximately [3]:

𝜓_0_ = 𝜁(1+𝐷/𝑎_1_)𝑒^𝜅𝐷^ (S3)

Where *ρ* is the surface charge density, 𝑘 is the Boltzmann constant, T is the absolute temperature, *ε* is the relative medium constant, *ε_0_* is the vacuum dielectric constant, n is the number of electrolytes per unit volume, e_0_ is the electron charge, z is the electrolyte valence, 𝜓_0_ is the surface potential, 𝑎_1_ is the particle stokes radius, 𝜁 stands for the zeta potential, 𝜅^−1^ stands for the Debye length, D is the distance from sliding layer to particle surface, respectively. The above formula indicates that the surface charge is determined by the zeta potential because other parameters are constant for PCN with different structure in this work.

**S4. DFT computational details**

The electronic structures for UPCN and SS-UPCN-375 were studied via DFT, where all geometries were optimized by PBE01 functional and def2-SVP2, 3 basis set by Gaussian 16. C02 quantum chemical package4 [4]. The harmonic frequencies were performed at the same level to confirm that all studied structures as minima possesses zero frequency, i.e. they are located at the minima at the potential energy surfaces [5]. The electrostatic potential (ESP) for complex was plotted by Gauss View, whose input file was extracted from Gaussian formatted checkpoint file [6, 7].

**Supplementary Figures**


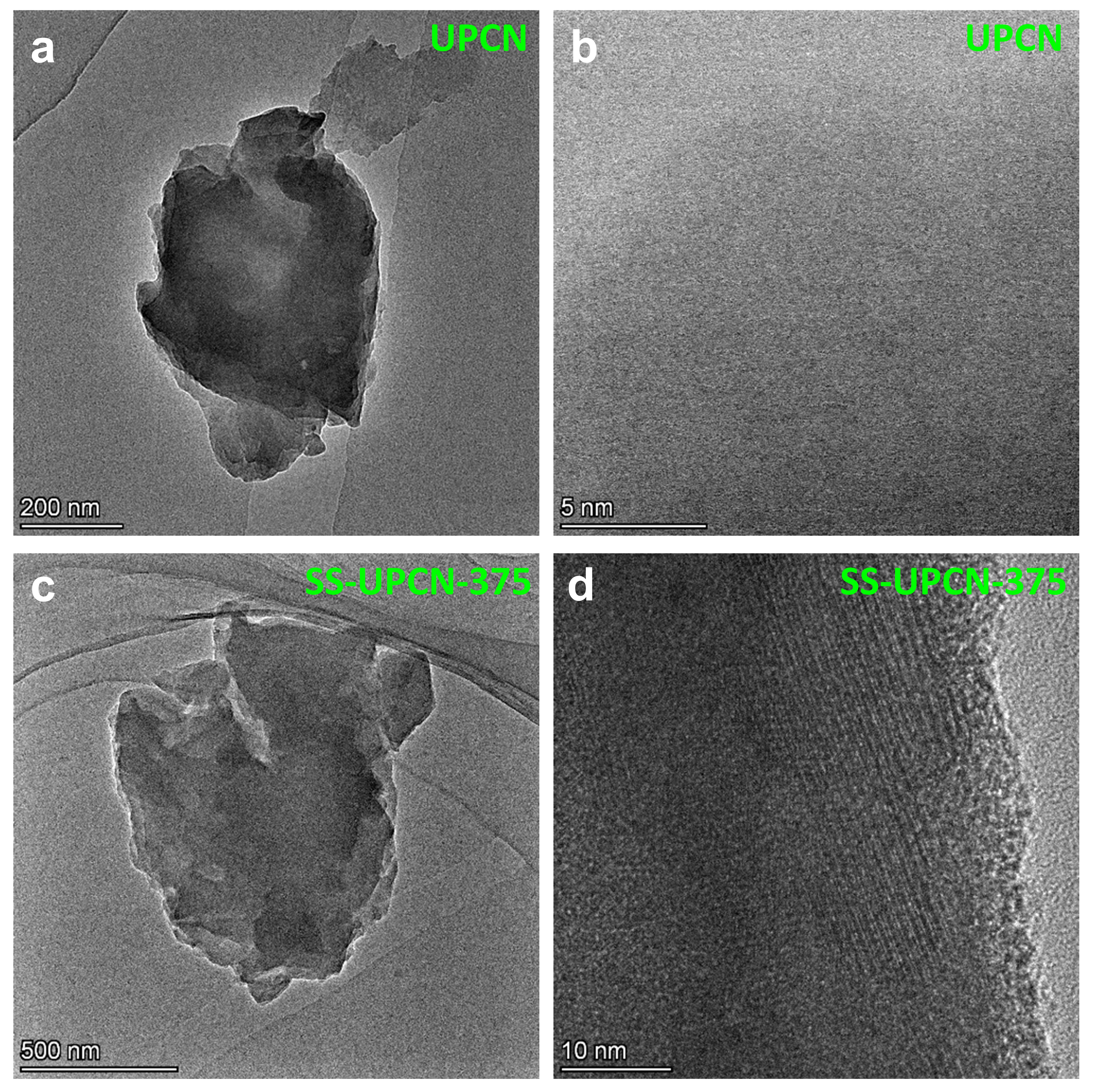


**Fig. S1** TEM and HR-TEM images of (a-b) original UPCN and (c-d) SS-UPCN-375.


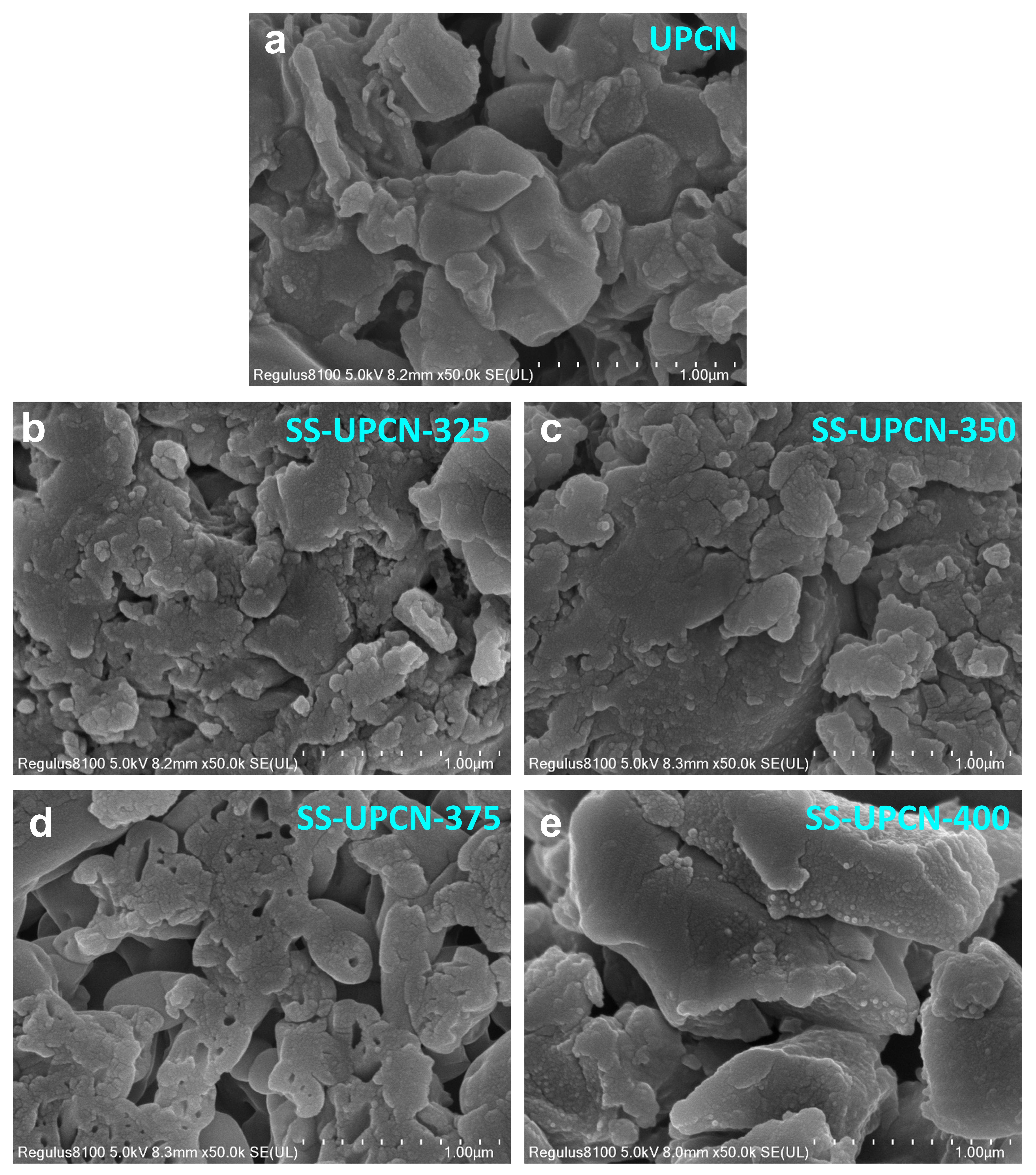


**Fig. S2** SEM images of (a) original UPCN and (b–e) the synthesized SS-UPCN-T.


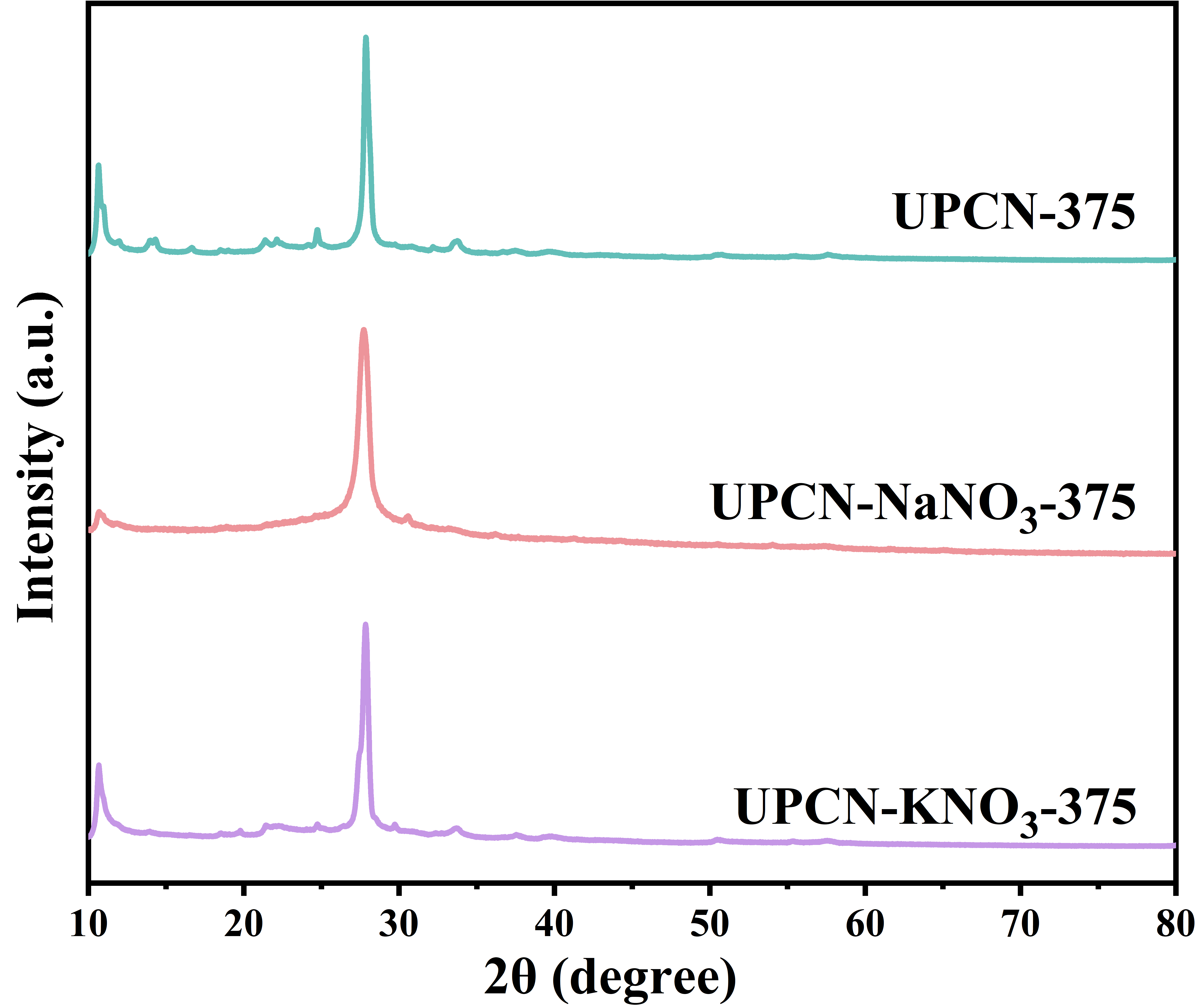


**Fig. S3** XRD patterns of UPCN-KNO_3_-375, UPCN-NaNO_3_-375 and UPCN-375 samples, respectively.





**Fig. S4** XPS of UPCN and SS-UPCN-375 samples: (a) Survey spectra; (b) C1s spectra and (c) N1s Spectra.


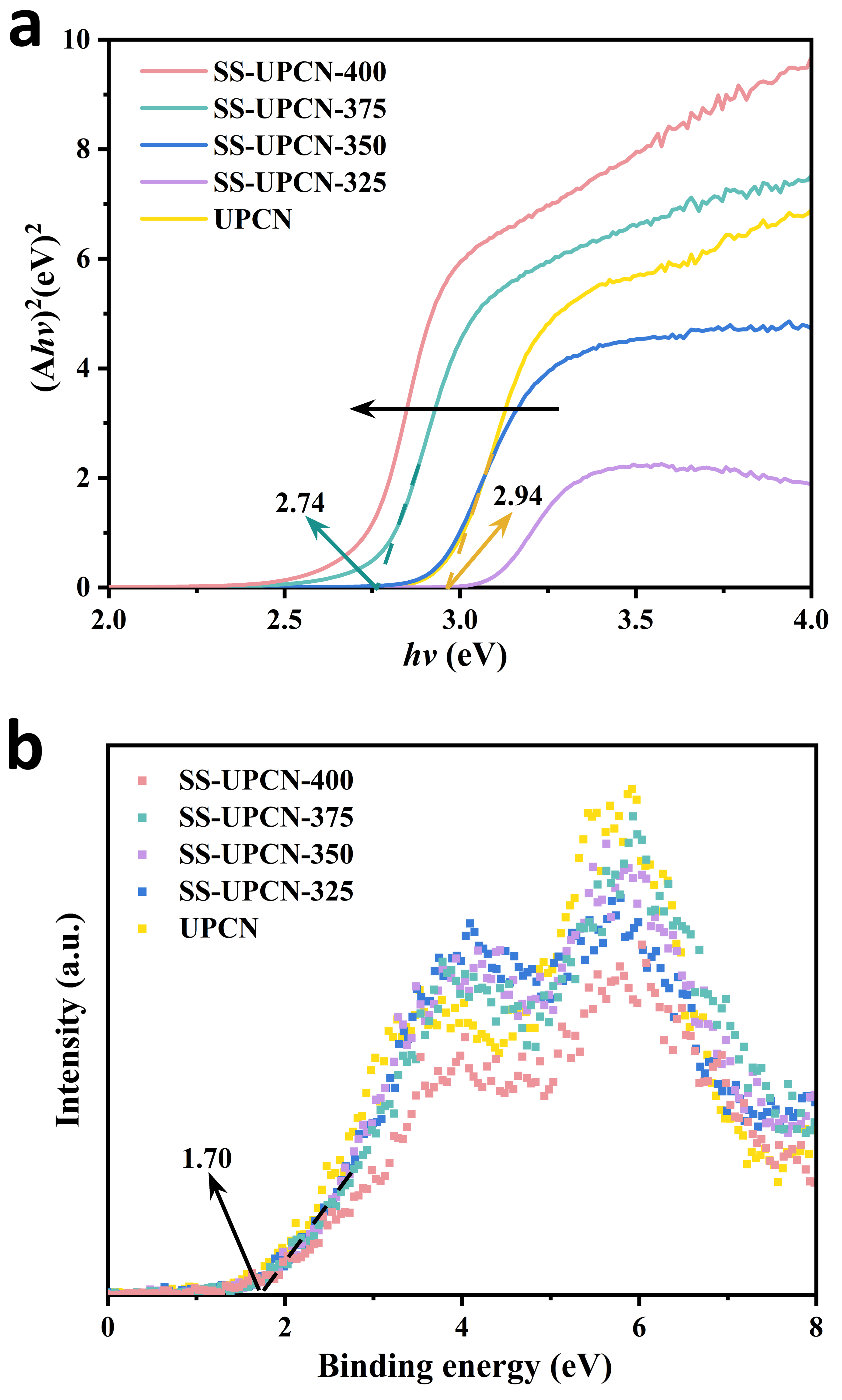


**Fig. S5** (a) Tauc plots and (b) VB-XPS spectra of UPCN and SS-UPCN-T samples.


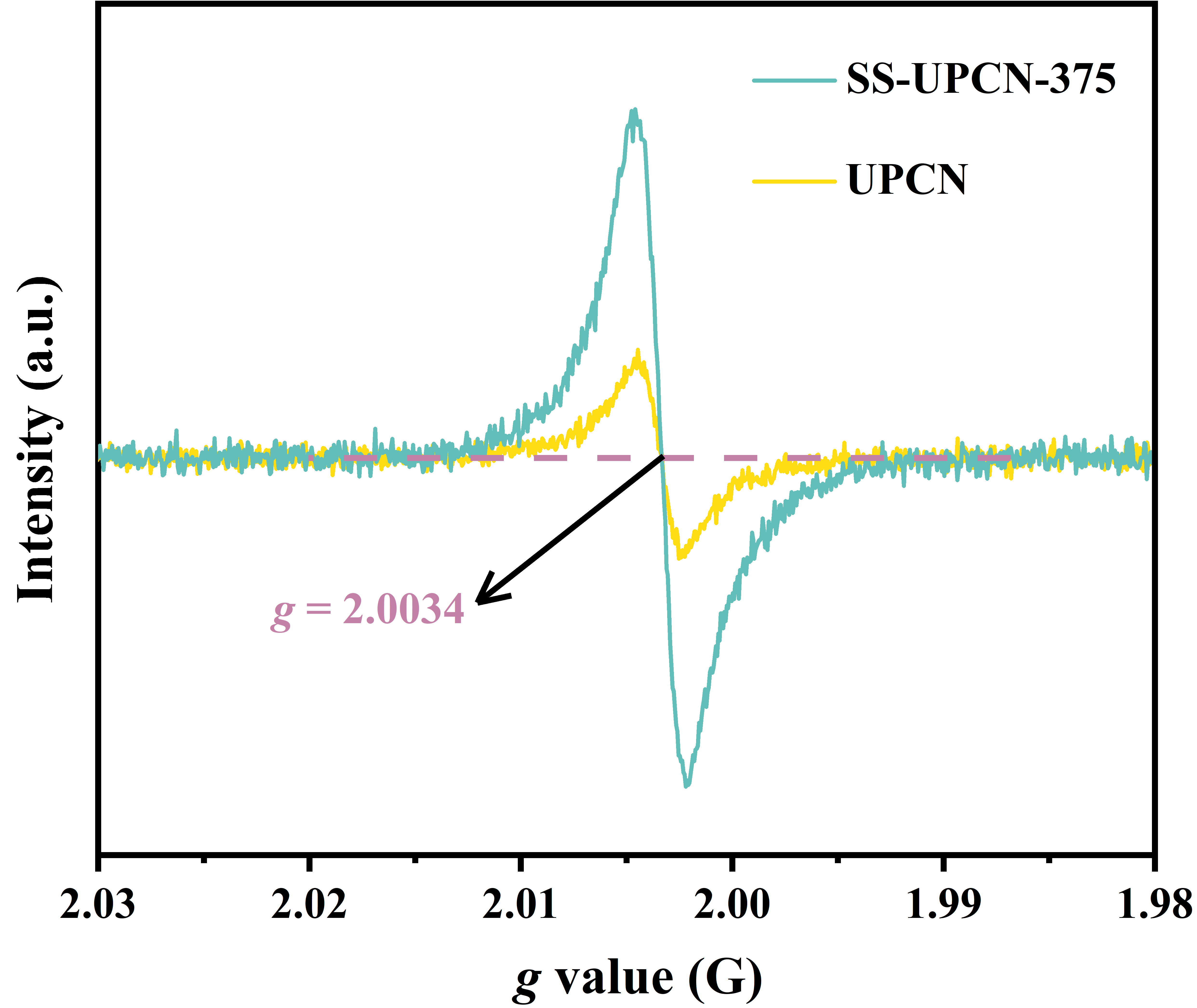


**Fig. S6** EPR spectra UPCN and SS-UPCN-375 samples.


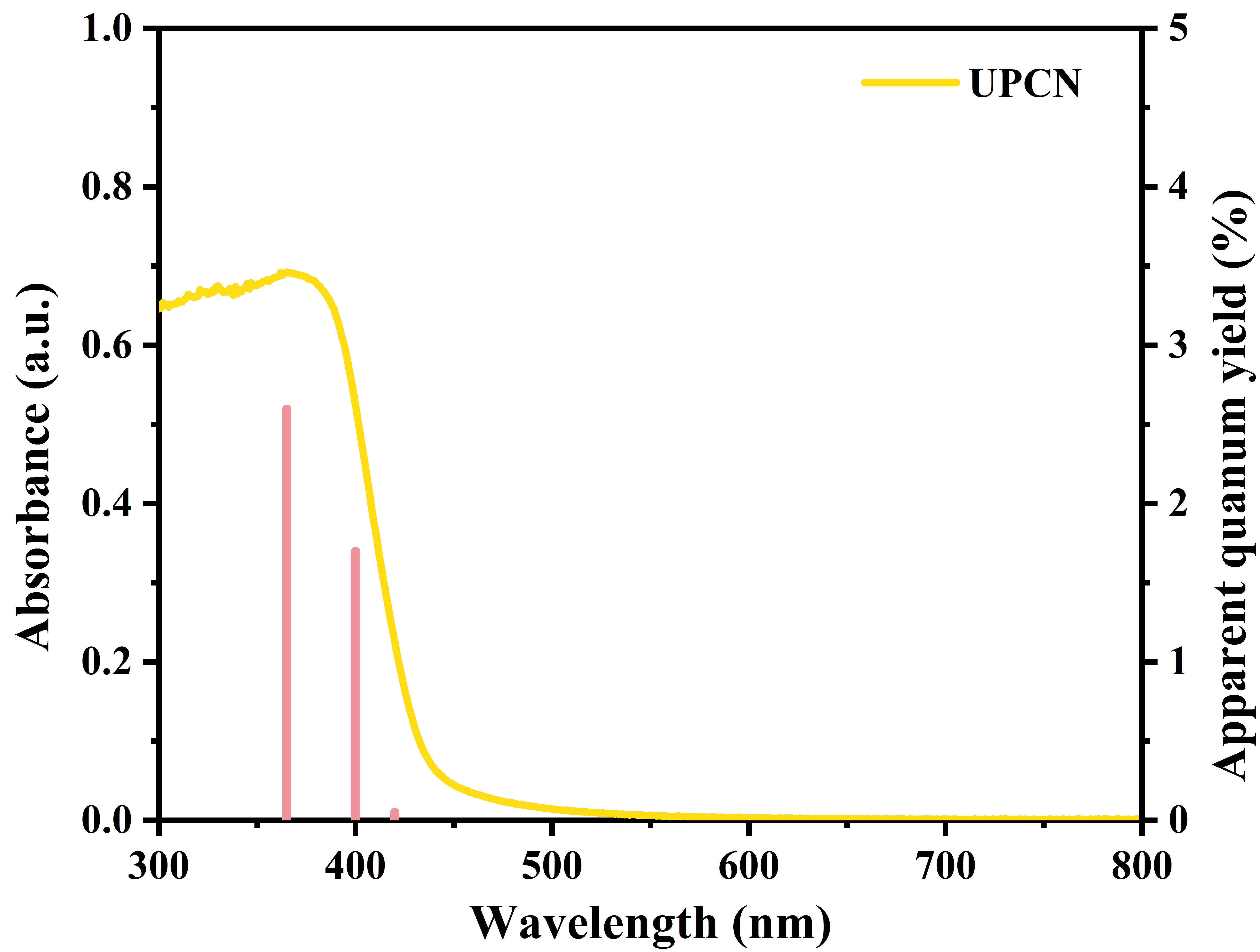


**Fig. S7** The AQY of H_2_O_2_ produced on UPCN sample.


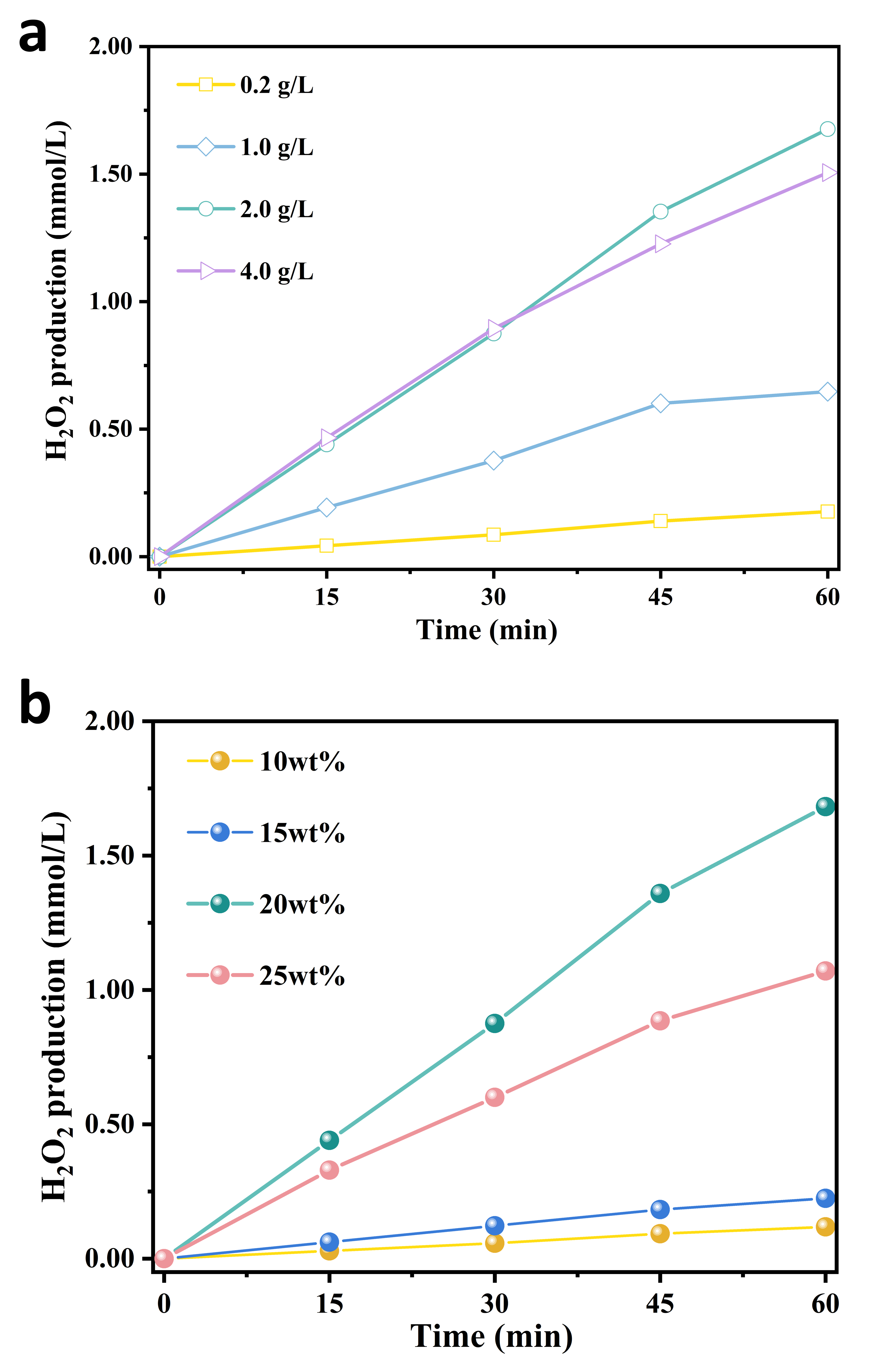


**Fig. S8** H_2_O_2_ production on SS-UPCN-375 sample with (a) different catalyst concentrations, (b) different mass ratios of m_solar salt/urea_.


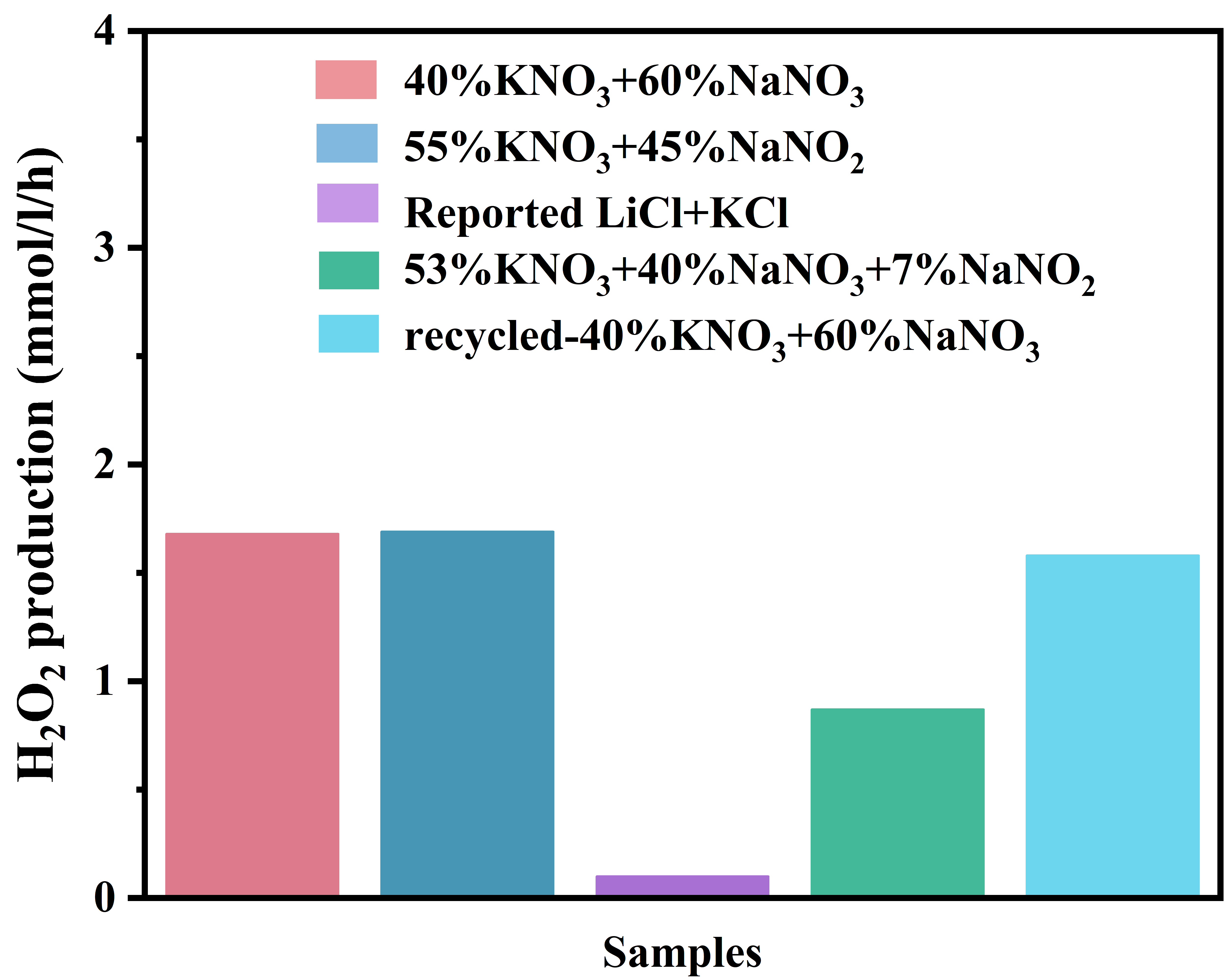


**Fig. S9** H_2_O_2_ production on PCN-375 samples under different molten salt treatment.


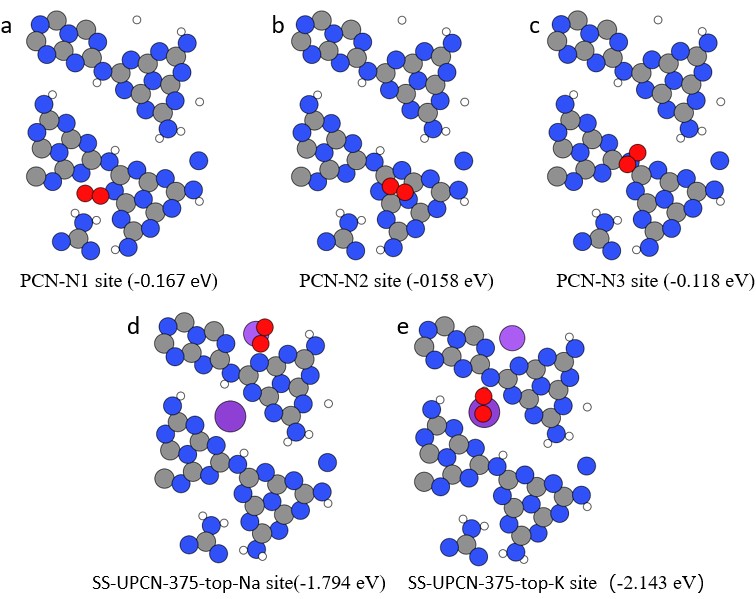


**Fig. S10** The adsorption energy of O_2_ at different sites in (a-c) PCN and (d, e) SS-UPCN-375.


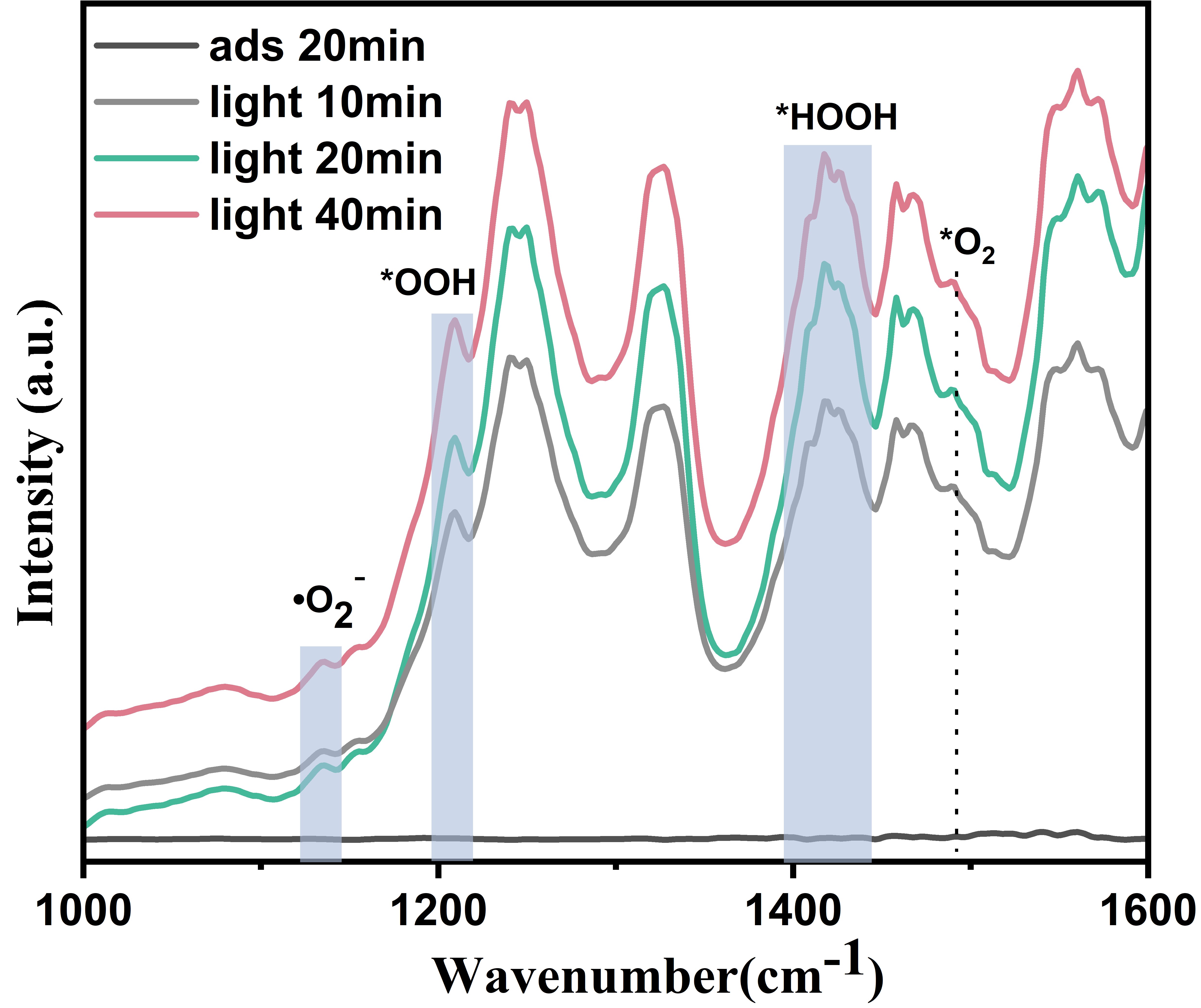


**Fig. S11** In situ FT-IR spectra of SS-UPCN-375 during the photocatalytic H_2_O_2_ production reaction processes.

**Tab. S1** Composition analysis of UPCN and SS-UPCN-T samples.


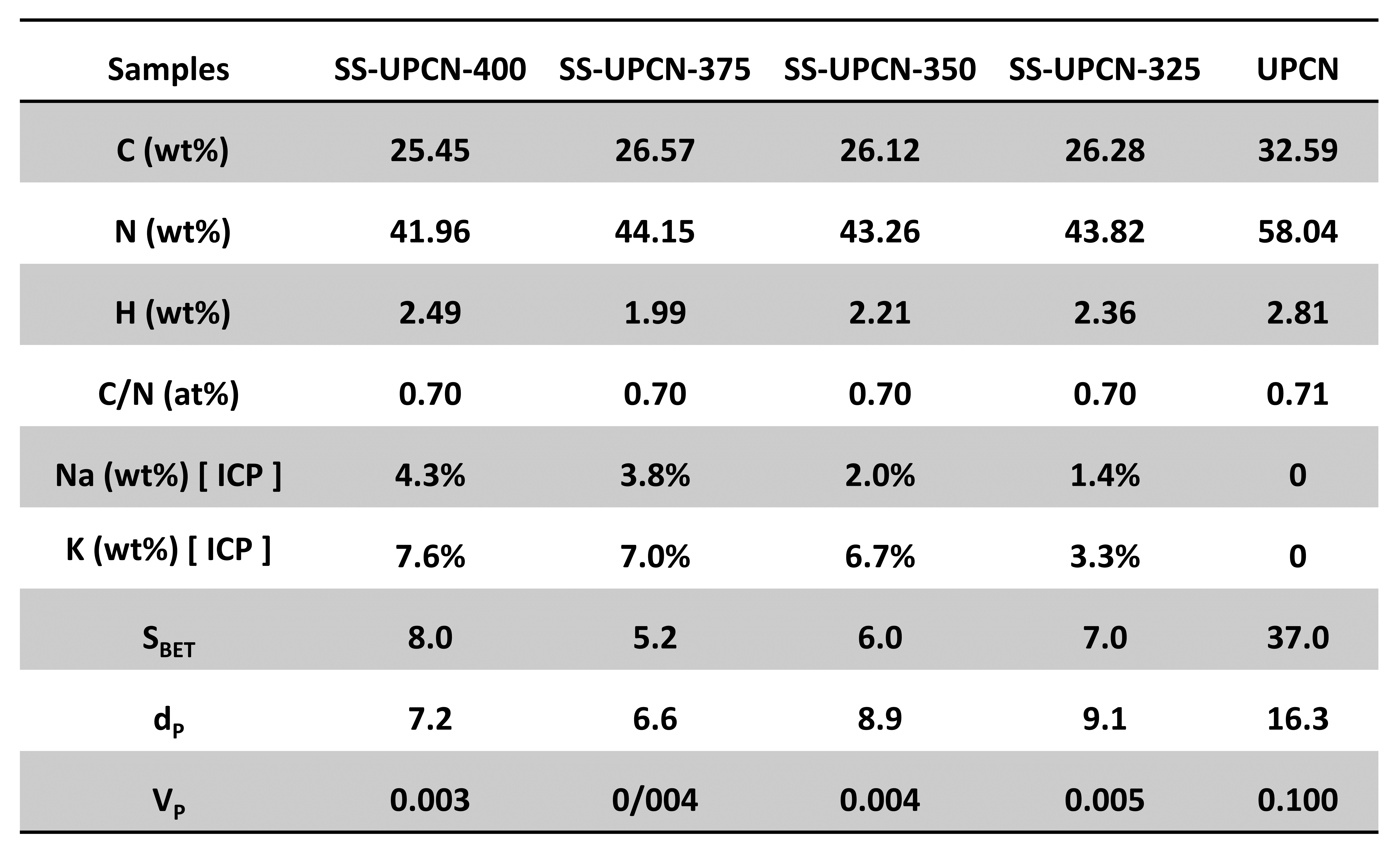


**Tab. S2** Calculated band structures of UPCN and SS-UPCN-T samples.


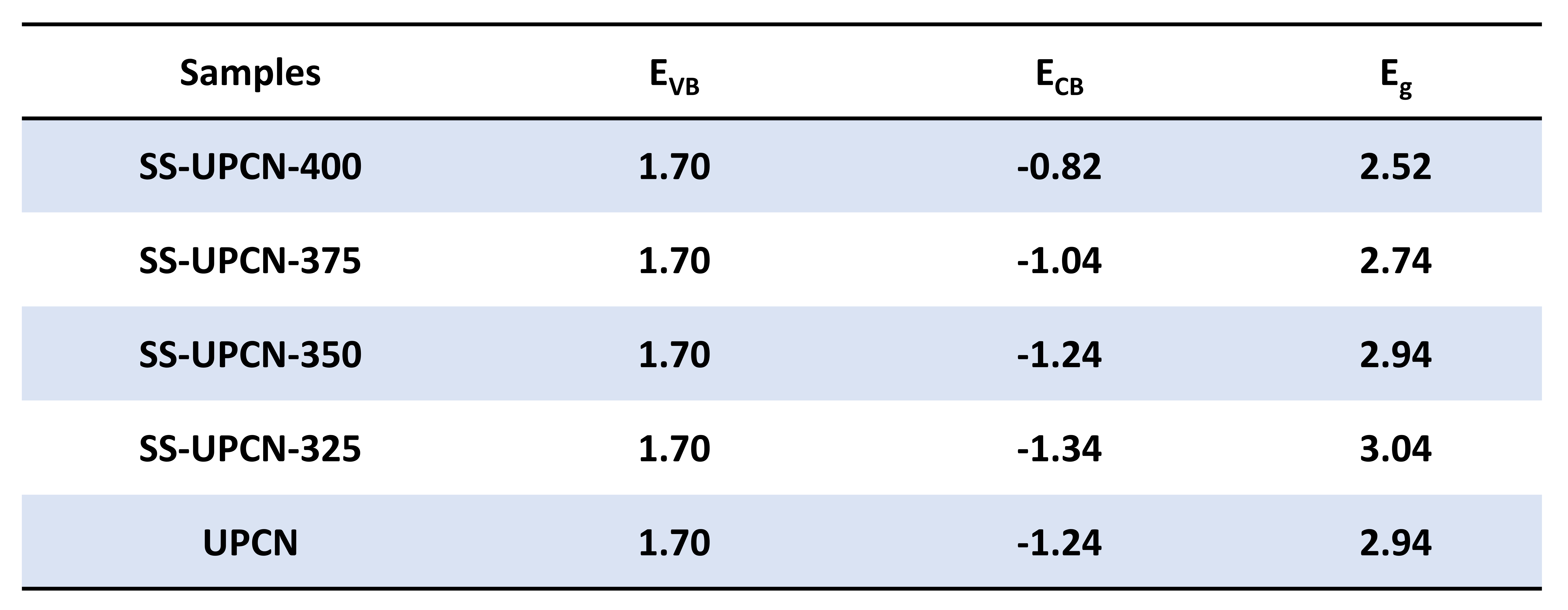


**Tab. S3** Comparison of SS-UPCN-375 with other photocatalysts for the photocatalytic H_2_O_2_ evolution rate.


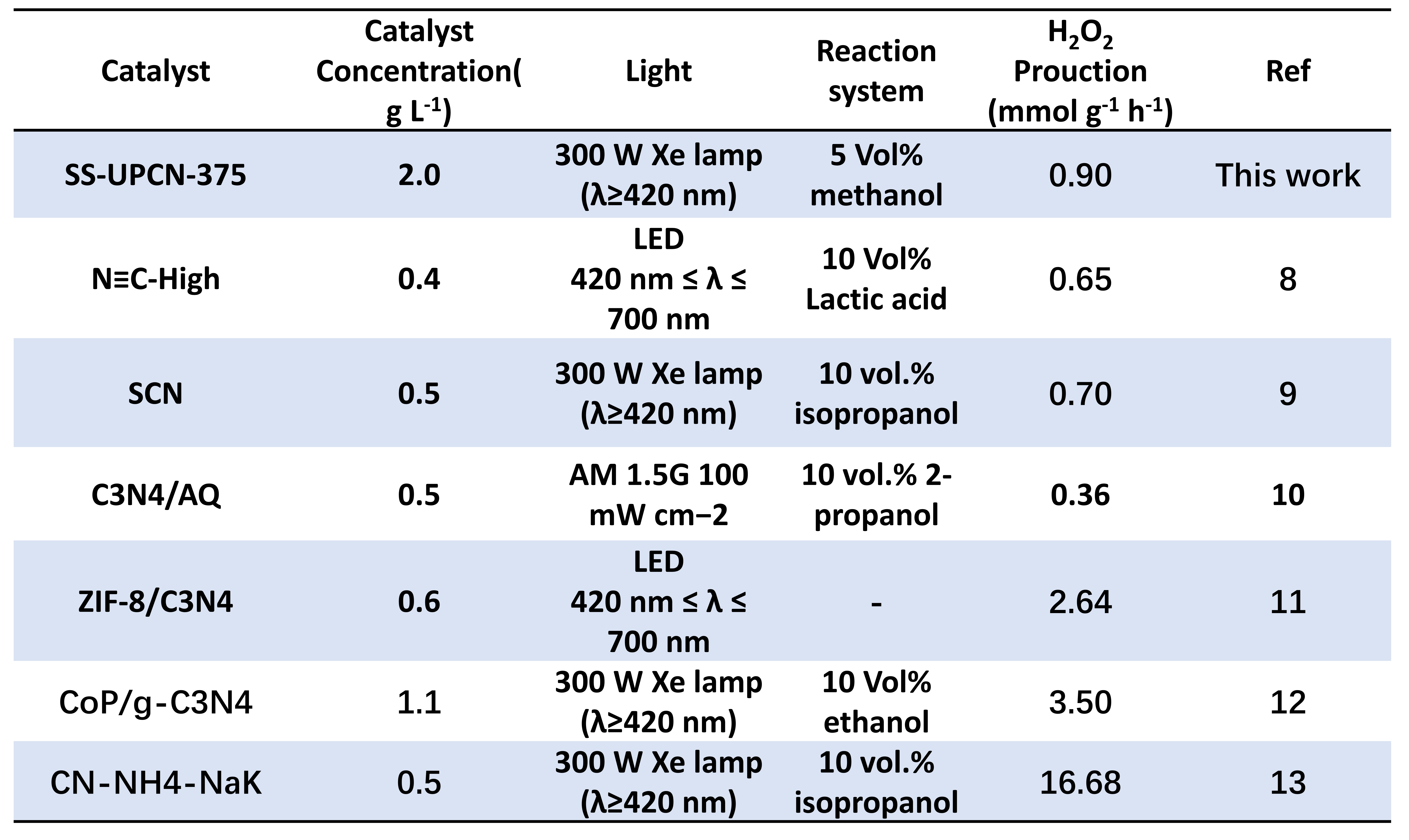


**References**

[1] J. Li, G. Zhan, Y. Yu, L. Zhang, *Nat. Commun.* **2016**, 7, 11480.

[2] J. Li, Y. Yu, L. Zhang, *Nanoscale* **2014**, 6, 8473.

[3] Y. Guo, W. Shi, Y. Zhu, Y. Xu, F. Cui, *Appl. Catal. B-Environ.* **2020**, 262, 118262.

[4] P. Lefebvre, J. Allègre, B. Gil, H. Mathieu, N. Grandjean, M. Leroux, J. Massies, P. Bigenwald, *Phys. Rev. B.* **1999**, 59, 15363.

[5] G. Kresse, J. Furthmüller, *Comp. Mater. Sci.* **1996**, 6, 15.

[6] G. Kresse, D. Joubert, *Phys. Rev. B.* **1999**, 59, 1758.

[7] J. P. Perdew, K. Burke, M. Ernzerhof, *Phys. Rev. Lett.* **1996**, 77, 3865.

[8] Y. Ma, H. Sun, Q. Wang, L. Sun, Z. Liu, Y. Xie, Q. Zhang, C. Su, D. Fan, *Appl. Catal. B-Environ.* **2023**, 335, 122878.

[9] C. Chu, W. Miao, Q. Li, D. Wang, Y. Liu, S. Mao, *Chem. Eng. J.* **2022**, 428, 132531.

[10] H.-i. Kim, Y. Choi, S. Hu, W. Choi, J.-H. Kim, *Appl. Catal. B-Environ.* **2018**, 229, 121.

[11] Y. Zhao, Y. Liu, J. Cao, H. Wang, M. Shao, H. Huang, Y. Liu, Z. Kang, *Appl. Catal. B-Environ.* **2020**, 278, 119289.

[12] Y. Peng, L. Wang, Y. Liu, H. Chen, J. Lei, J. Zhang, *Eur. J. Inorg. Chem.* **2017**, 2017, 4797.

[13] F. He, Y. Lu, Y. Wu, S. Wang, Y. Zhang, P. Dong, Y. Wang, C. Zhao, S. Wang, J. Zhang, S. Wang, *Adv. Mater.* **2023**, 36, 2307490.
